# *GmNAP1* is essential for trichome and leaf epidermal cell development in soybean

**DOI:** 10.1007/s11103-020-01013-y

**Published:** 2020-05-15

**Authors:** Kuanqiang Tang, Suxin Yang, Xingxing Feng, Tao Wu, Jiantian Leng, Huangkai Zhou, Yaohua Zhang, Hui Yu, Jinshan Gao, Jingjing Ma, Xianzhong Feng

**Affiliations:** 1 Key Laboratory of Soybean Molecular Design Breeding, Northeast Institute of Geography and Agroecology, The Innovative Academy of Seed Design, Chinese Academy of Sciences, Changchun, 130102 Jilin China; 2 University of Chinese Academy of Sciences, Beijing, 100049 China

**Keywords:** Soybean, Trichome, Pavement cell, *GmNAP1*

## Abstract

**Key message:**

Map-based cloning revealed that two novel soybean distorted trichome mutants were due to loss function of *GmNAP1* gene, which affected the trichome morphology and pavement cell ploidy by regulating actin filament assembly.

**Abstract:**

Trichomes increase both biotic and abiotic stress resistance in soybean. In this study, *Gmdtm1-1* and *Gmdtm1-2* mutants with shorter trichomes and bigger epidermal pavement cells were isolated from an ethyl methylsulfonate mutagenized population. Both of them had reduced plant height and smaller seeds. Map-based cloning and bulked segregant analysis identified that a G-A transition at the 3ʹ boundary of the sixth intron of *Glyma.20G019300* in the *Gmdtm1-1* mutant and another G-A transition mutation at the 5ʹ boundary of the fourteenth intron of *Glyma.20G019300* in *Gmdtm1-2*; these mutations disrupted spliceosome recognition sites creating truncated proteins. *Glyma.20G019300* encodes a *Glycine max* NCK-associated protein 1 homolog (*GmNAP1*) in soybean. Further analysis revealed that the *GmNAP1* involved in actin filament assembling and genetic information processing pathways during trichome and pavement cell development. This study shows that *GmNAP1* plays an important role in soybean growth and development and agronomic traits.

**Electronic supplementary material:**

The online version of this article (10.1007/s11103-020-01013-y) contains supplementary material, which is available to authorized users.

## Introduction

Plant cells exhibit a wide variety of shapes that make important contributions to organ and tissue development and morphogenesis (Smith and Oppenheimer 2005; Yanagisawa et al. 2015). Trichomes, pavement cells, and stomata are three important components of leaf epidermal cells and play pivotal roles at each stage of development (Hegebarth and Jetter 2017). Leaf epidermal pavement cells generally have an interlocking jigsaw-puzzle shape in dicots with no protrusions or gas-exchange abilities. They protect the tissue layers located underneath, ensuring that morphologically more specialized cells are spaced out correctly; it also provides mechanical strength while still allowing growth and flexibility, and protect plants via functions such as maintaining temperature and resisting foreign invasion (Glover 2000). Stomata and trichomes are morphologically specialized (Mauricio and Rausher 1997; Serna and Martin 2006). Some flowering plants, such as tobacco (*Nicotania tabacum*), produce multicellular trichomes, whereas others, such as Arabidopsis, have unicellular trichomes (Glover 2000). Trichomes exist on many aerial plant parts, including leaves, stems, and sepals (Huchelmann et al. 2017; Liang et al. 2014), and help to protect the plant against herbivores and insects, deter microorganisms, and maintain ion homeostasis (Schilmiller et al. 2008); for example, the trichomes in strawberry plants act as a physical barrier creating difficulties for *Chaetosiphon fragaefolii* to feed (Benatto et al. 2018). Many previous studies have proved that trichomes play an efficient role in reducing water loss through decreasing the rate of transpiration, on account of their barrier effect against CO_2_ and H_2_O exchange (Fu et al. 2013; Ning et al. 2016). Trichomes can also prevent the field spread of soybean mosaic virus (Ren et al. 2000) and increase resistance to lepidopteran insects (Hulburt et al. 2004). Flavonoid aglycones or highly methylated flavonoids biosynthesized in the trichomes also provide a chemical barrier against highly energetic and deeply penetrating UV wavelengths (Hegebarth and Jetter 2017; Oliveira and Penuelas 2002; Tattini et al. 2005).

In soybean, the surfaces of leaf, stem, petiole and pod are covered with trichomes, and they play an important role in biological and abiotic stress, such as drought tolerance (Du et al. 2009a) and pest resistance (Chang and Hartman 2017; Ortega et al. 2016). There are many soybean mutants that have been described and collected in the USDA NIL collection (Bernard et al. 1991; Bernard and Singh 1969). Bernard and Singh (Bernard and Singh 1969) reported that five loci control the different kinds of aberrant trichome phenotypes of soybean, including *P1* (glabrous), *pc* (curly pubescence), *Pd* (puberulent density), *Ps* (puberulent sparse) and *p2* (puberulent). *Pd1* (puberulent density1) and *Pd2*, have been identified to control the trichome density of soybean (Pfeiffer and Pilcher 2006). More than 50 QTLs associated with trichome related traits have been identified in soybean (Chang and Hartman 2017; Du et al. 2009b; Fang et al. 2017; Komatsu et al. 2007; Oki et al. 2011; Sonah et al. 2015; Vuong et al. 2015). *T* locus encodes a flavonoid 3′-hydroxylase (F3′H) that controls the trichome color (Toda et al. 2002; Zabala and Vodkin 2003).

The SCAR/WAVE (suppressor of cAMP receptor/WASP family verpro lin-homologous) complex has been shown to be the major nucleator of actin filament networks in plants (Guimil and Dunand 2007; Qian et al. 2009). SCAR/WAVE proteins form a pentameric complex containing Abi (Abl-interactor), NAP (Nck-associated protein), PIR121 (p53-inducible mRNA 121), and HSPC300 (haematopoietic stem progenitor cell 300). Many mutants of the SCAR/WAVE complex have been identified in Arabidopsis, such as *grl* (*gnarled*)/*nap1* (El-Assal Sel et al. 2004), *pir1* (*pirogi*) (Li et al. 2004), *sra1* (*specifically rac1-associated protein 1*) (Basu et al. 2004), *dis3* (*distorted1*) (Basu et al. 2005), *brk1* (*brick1*) (Folkers et al. 2002), and *spk1* (*spike1*) (Qiu et al. 2002). Most mutations lead to swelling and reduce branch length of trichomes and loss of interdigitation and gaps between adjacent pavement cells, and WAVE complexes are unstable in the absence of any of their members (Qian et al. 2009). Because the SCAR/WAVE complex is considered to be the only regulator of ARP2/3 (Actin-Related Protein 2/3), some mutants of the ARP2/3 complex in Arabidopsis, such as *arp2* (Li et al. 2003), *arp3/dis1* (Li et al. 2003), *dis2* (El-Din El-Assal et al. 2004), and *crk* (*cysteine-rich receptor-like kinase*) (Li et al. 2003), also display very similar phenotypes in pavement cells and trichomes to those of the “distorted” mutants of the SCAR/WAVE complex.

Campbell et al. (2016) identified a fast neutron-induced the gnarled trichrome mutant and mapped a 26.6 megabase interval on chromosome 20 that co-segregated with the mutant phenotype. The chromosome 20 interval included a small structural variant within the coding region of a soybean ortholog (*Glyma.20G019300*) of *Arabidopsis* Nck-Associated Protein 1 (NAP1). A wild-type soybean NAP1 transgene functionally complemented an *Arabidopsis nap1* mutant. They also proved that a historic spontaneous soybean gnarled trichrome mutant (T31) identified a frame shift mutation resulting in a truncation of the coding region of *Glyma.20G019300*. This work shows that mutation of *NAP1* locus result in gnarled trichomes, however, further molecular and cellular evidence still needed to reveal its function of *GmNAP1* in trichome development.

In this study, two novel *Glycine max distorted trichome mutant 1–1* and *1–2* (*Gmdtm1-1* and *1-2*) were characterized with visibly smoother leaf, and genetic mapping proved that *GmNAP1* mutations cause abnormal trichome and pavement cell development in above two mutants. The transcriptional profile analysis demonstrated *GmNAP1* gene involved in actin filament assembling and genetic information processing pathways during trichome development. We further show that abnormal trichome shape and pavement size in *Gmnap1* mutation involved the F-actin density in the trichome tip and the pavement cell ploidy, separately.

## Result

### Abnormal development of trichomes and pavement cells in *Gmdtm1-1* and *Gmdtm1-2* mutants

**Fig. 1:**
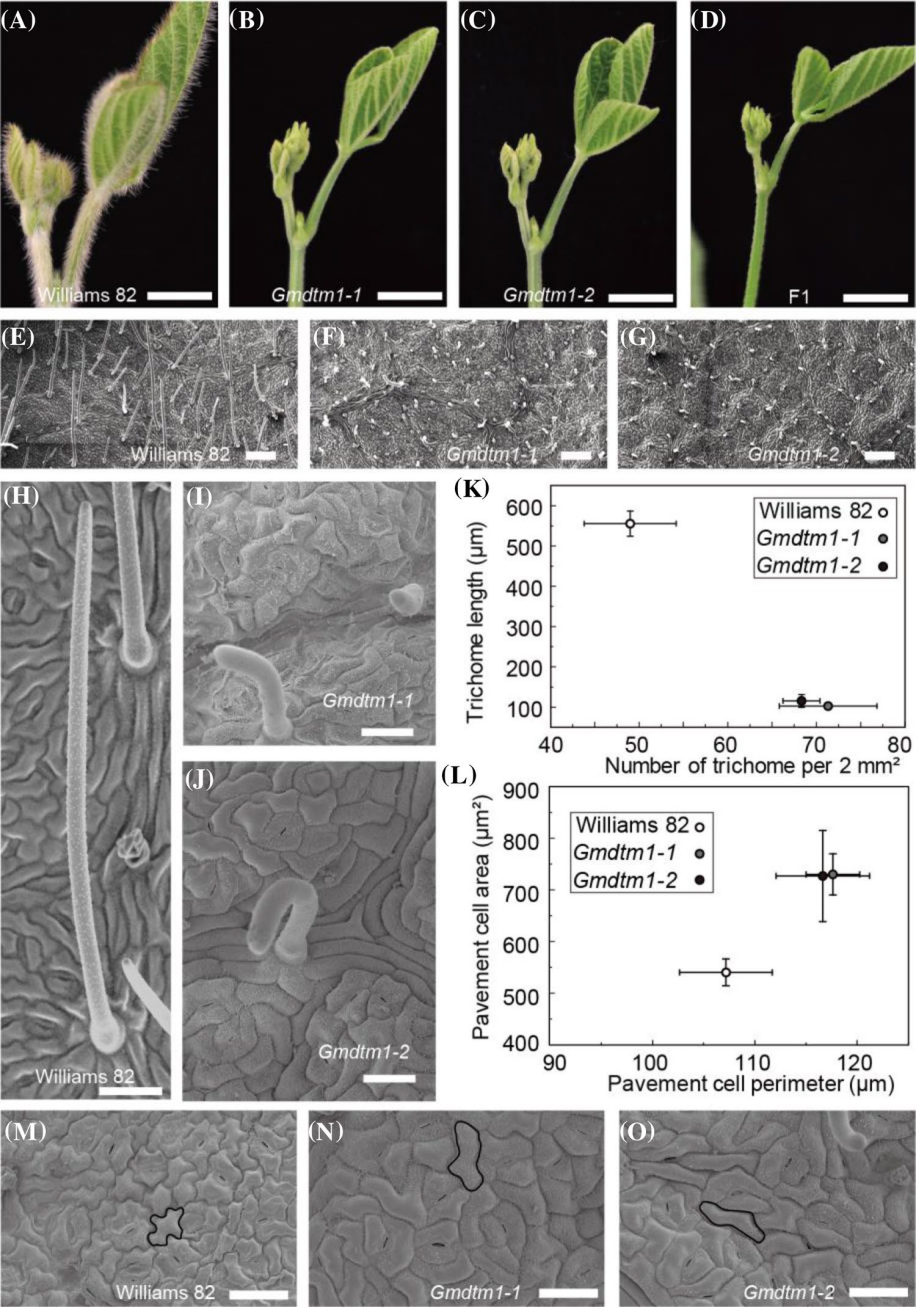
**a**–**c** Young leaves phenotype at V2 stage when the second trifoliolate was fully opened, Bars = 1 cm. **d** F1 plants of *Gmdtm1-1* × *Gmdtm1-2* at V2 stage. Bar = 1 cm. **e**–**g** SEM images of leaf surface. Bar = 0.2 mm. **h**–**j** SEM images of trichome shape. Bar = 100 μm. **k** Leaf trichome density and length. **l** Epidermal pavement cell area and perimeter length of leaf. **m**–**o** SEM images of leaf pavement cells. Bars = 50 μm. Dark lines indicate representative cells

Sixteen leaf surface related mutants were obtained from EMS mutant population in our laboratory as previous described (Feng et al. 2019; Gao et al. 2017). Nine of them with hair color change, five of them with glabrous leaf, and two of them with more hair. Two of five of glabrous leaf mutants, *Gmdtm1-1* and *Gmdtm1-2*, were studied in this paper. In contrast to the wild type plant fully covered with trichome in the young leaf and stem (Fig. 1a), these two mutants had smaller glabrous leaf and stem (Fig. 1b, c). In order to investigate the genetic relationship of *Gmdtm1-1* and *1-2*, they were crossed to each other, and their F1 progeny also had same phenotype as their parents. It indicated that *Gmdtm1-1* and *Gmdtm1-2* were allelic to each other (Fig. 1d).

The trichomes of leaves were usually straight with sharp tips in wild-type (Fig. 1h). However, the trichomes of the two mutants were not erected but drooping and had blunt tips (Fig. 1f, g, i, j). The length of the trichomes in *Gmdtm1-1* and *1-2* were about 81% and 79% shorter than that of wild-type trichomes, respectively (Fig. 1h–k). The trichomes number per 2 mm^2^ in *Gmdtm1-1* and *Gmdtm1-2* were 46% and 39% more than those in wild-type respectively (Fig. 1e, f, k). These results illustrated that trichome development in *Gmdtm1-1* and *1-2* was dramatically different from wild-type plants in terms of size, density, and shape.

Epidermal pavement cells of wild-type Williams 82 plants were arranged in a jigsaw-puzzle pattern (Fig. 1m). The jigsaw-puzzle appearance of epidermal pavement cells was less apparent in the two alleles, *Gmdtm1-1* and *1-2*, than in the wild type (Fig. 1n, o). The area and perimeter length of epidermal pavement cells in *Gmdtm1-1* and *Gmdtm1-2* were increased comparing with Williams 82 (Fig. 1l). The pavement cell area of *Gmdtm1-1* and *Gmdtm1-2* was increased by 34.54% and 35.13% respectively compared with Williams 82 (see above) (Fig. 1l). In addition, the perimeter length of pavement cells of *Gmdtm1-1* and *Gmdtm1-2* displayed a 8.83% and 9.76% increase over than that observed in Williams 82 (see above) (Fig. 1l). These results indicate that the phenotypes of epidermal pavement cells in *Gmdtm1-1* and *Gmdtm1-2* were also affected.

### The *Gmdtm1-1* mutation was mapped to *Glyma.20G019300* gene

To understand the inheritance pattern of *Gmdtm1-1*, we crossed *Gmdtm1-1* with ‘Hedou 12’. The F1 plants showed a similar phenotype to the wild type, indicating that the *Gmdtm1* mutation is recessive. Of 334 F2 plants analyzed, 86 showed the *Gmdtm1* mutant phenotype. The ratio of the wild type and mutant type in the F2 population corresponded to the expected 3:1 segregation ratio for a single recessive gene (*χ*^*2*^ test, *p* = 0.82), indicating that the defect in *Gmdtm1-1* behaved in a monogenic recessive manner.

**Fig. 2:**
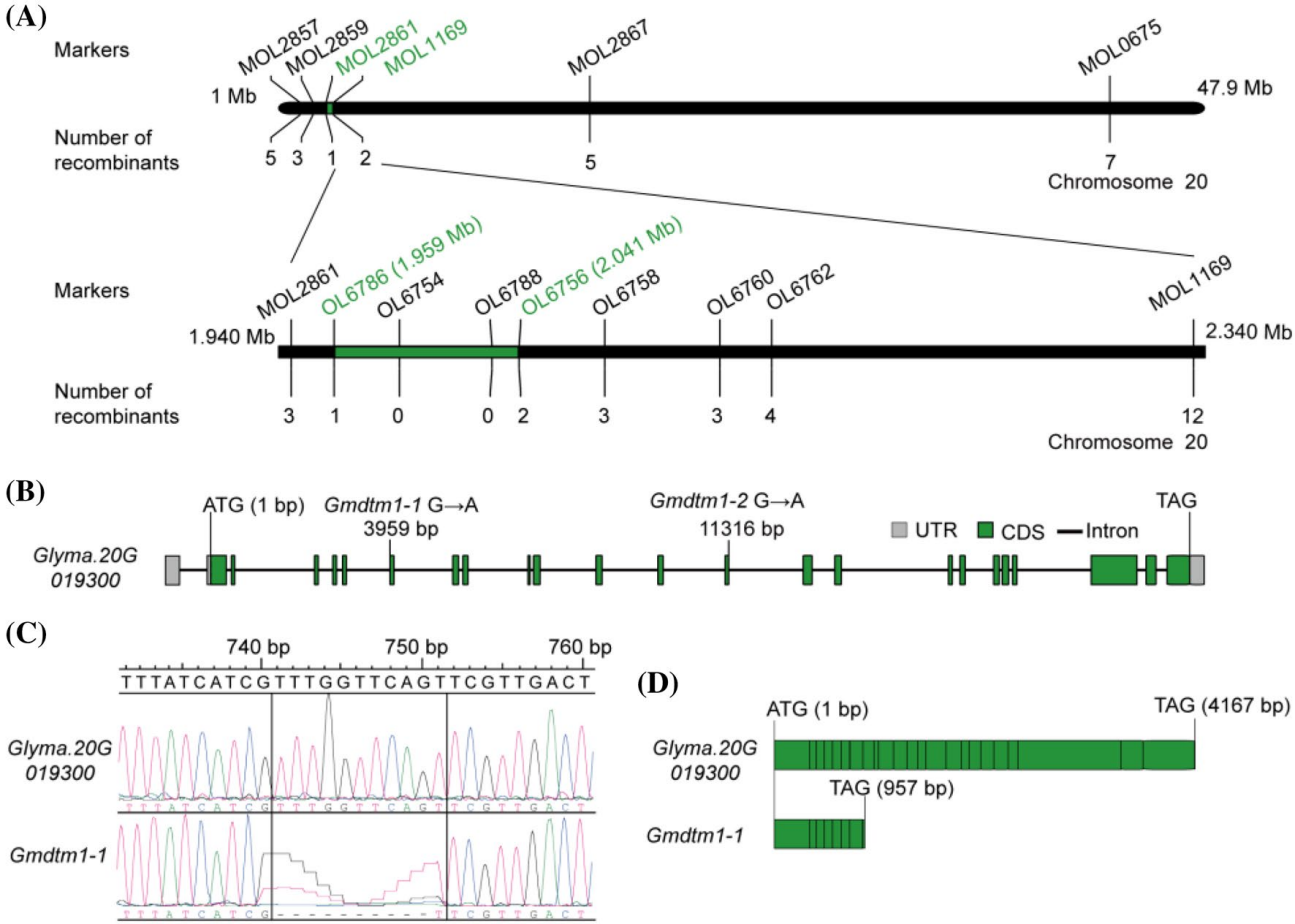
Positional cloning and characterization of the *Gmdtm1* locus. **a** Mapping of the *Gmdtm1* locus. The *Gmdtm1* locus was delimited to a 0.4 Mb region (in green color) between InDel markers MOL2861 and MOL1169 on chromosome 20 and further to an 82 kb region (in green color) bounded by markers OL6786 and OL6756. **b** Schematic structure of *Glyma.20G019300* gene and mutant alleles. **c** A 10 bp deletion of *Gmdtm1-1* transcript comparing with *Glyma.20G019300* transcript.** d** The predicted CDS length of *Glyma.20G019300* and *Gmdtm1-1* was 4167 and 957 bp, respectively

To locate the *Gmdtm1* locus, we used approximately 165 InDel markers between ‘Hedou 12’ and Williams 82 for mapping. The *Gmdtm1-1* locus was delimited to a 0.4 Mb region between InDel markers MOL2861 (1.940 Mb) and MOL1169 (2.340 Mb) on chromosome 20 (Fig. 2a). Fifteen recombinants for the markers MOL2861 (1.940 Mb) or MOL1169 (2.340 Mb) among the F2 plants were used for further fine mapping. The *Gmdtm1* locus was further pinpointed to a 0.082 Mb region between markers OL6786 (1.959 Mb) and OL6756 (2.041 Mb), containing seven annotated genes according to the Williams 82 reference genome (*Glycine max Wm82.a2.v1*) (Fig. 2a, Table S1). Sequencing of the 82 Kb genomic DNA region containing these seven genes revealed that only *Glyma.20G019300* gene had a G-to-A change in 3959 bp between Williams 82 and *Gmdtm1-1* mutant, while no sequence difference was detected in the other six genes. Transcripts analysis of *Glyma.20G019300* gene indicated that there was a 10 bp deletion in the seventh exon of *Gmdtm1-1* mutant comparing Williams 82 (Fig. 2b, c). Further analysis indicated that the G-to-A change of *Glyma.20G019300* disrupted the splice acceptor site and created a new splicing acceptor site at 10 bp downstream of the mutation site. In addition, the 10 bp deletion in the CDS resulted in a frameshift and a premature stop codon to produce a putative truncated protein lacking 1133 amino acid residues of the carboxyl region (Fig. 2c, d). This mutation of *Glyma.20G019300* may be responsible for the phenotype of *Gmdtm1-1*.

### The *Gmdtm1-2* mutation was also mapped to *Glyma.20G019300* gene

Another distorted trichome mutant, *Gmdtm1-2*, was discovered from the same EMS mutagenesis population as above. The morphology of *Gmdtm1-2* was very similar to that of *Gmdtm1-1*. Genetic analysis indicated that there were 89 mutant plants segregating from 289 progeny plants of the heterozygous *Gmdtm1-2* plants. The ratio of the mutant and wild type in this population was in accordance with the expected 1:3 distribution. This indicates that *Gmdtm1-2* is a single recessive mutant.

**Fig. 3:**
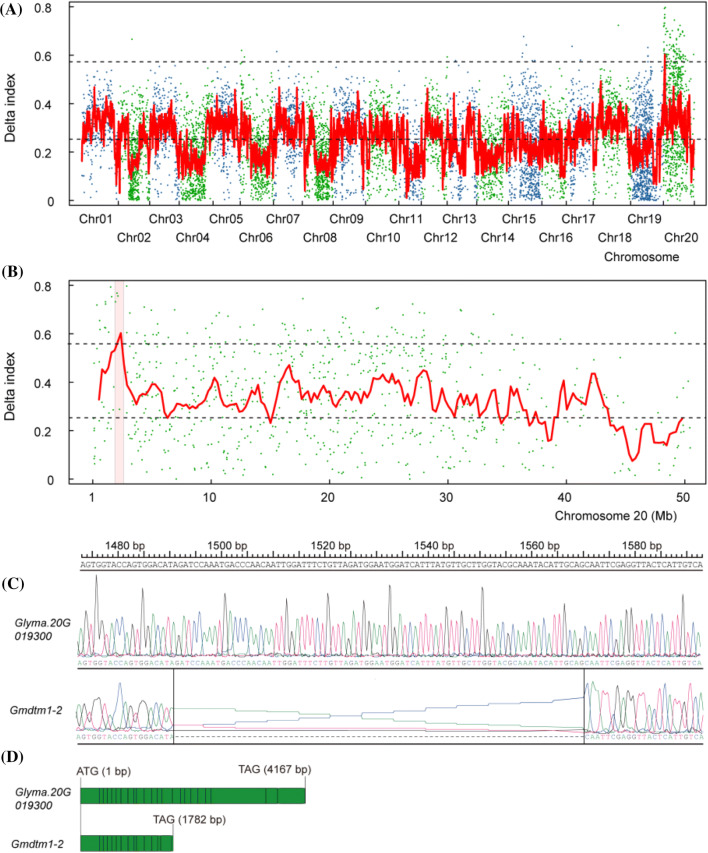
Bulked segregant analysis (BSA) mapping of *Gmdtm1-2*.** a** Delta SNP index plot over all chromosomes. Upper and lower dotted lines represent mean value + 4 × standerd error and mean value, respectively. **b** Delta SNP index plot on chromosome 20. The interval between 2 Mb and 2.5 Mb of chromosome 20 with a highest peak is the candidate region for *Gmdtm1-2*, in which the ΔSNP-index was greater than mean value + 4 × standerd error. **c** The different region between *Glyma.20G019300* transcript and the new transcript sequences of *Gmdtm1-2*. **d** The predicted CDS length of *Glyma.20G019300* and *Gmdtm1-2* was 4167 and 1782 bp, respectively

To investigate which gene contributed to the distorted trichome phenotype in *Gmdtm1-2*, we re-sequenced the genome of the mutant and wild-type pools from the *Gmdtm1-2* M2 segregating population and calculated a SNP index using the BSA method (see details in “Materials and methods” section). A total of 8866 SNPs were detected from the two pools after filtering and used to plot the chart. A linkage analysis with SNP index distribution revealed that the 0.5 Mb region between 2.0 Mb and 2.5 Mb on chromosome 20 co-segregated with the defective trichome phenotypes of *Gmdtm1-2* (Fig. 3a, b), while no other major divergence of allele frequencies detected between the two bulks in other chromosome. Only two SNP mutations were discovered in the candidate interval, a C to T transversion in 2,009,538 bp and a G to A transition in 2,134,337 bp of chromosome 20. The transition (G/A) located in the 5′UTR region of *Glyma.20G020800* and did not change its protein sequence. The transversion (C/T) located at the left boundary of the fourteenth intron of *Glyma.20G019300,* and led to a new transcript (Fig. 3c). Compared with *Glyma.20G019300* transcript of ‘Williams 82, the transcript of *Gmdtm1-2* has a 19 bp deletion and gave rise to a truncated protein (Fig. 3c, d). These results indicate that *Gmdtm1-2* phenotype is also caused by *Glyma.20G019300* mutation.

### *GmNAP1* encodes a NCK-associated protein 1

**Fig. 4:**
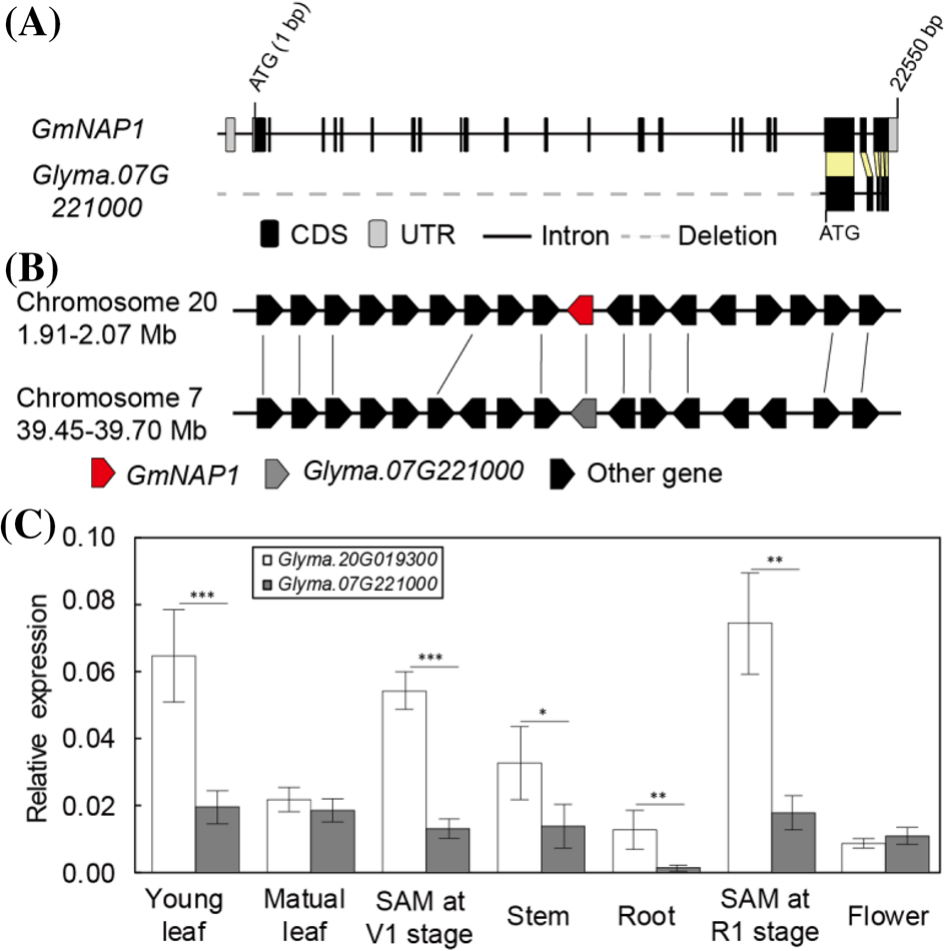
**a** Gene structure of *GmNAP1* and its ortholog, *Glyma.07G221000*, in soybean. **b** Syntenic plot of sequence assemblies surrounding *GmNAP1* and *Glyma.07G221000*. The red arrow represents the anchor *GmNAP1* gene, and the gray arrow represents *Glyma.07G221000*. The flanking genes around *GmNAP1* and *Glyma.07G221000* are indicated by black arrows. Conserved gene pairs between the segments are connected by lines. **c** Expression profiles of the *Glyma.20G019300* (*GmNAP1*) and its homologous gene, *Glyma.07G221000*. Asterisks indicate significant differences as determined by Student’s *t*-test (*p < 0.05; **p < 0.01; ***p < 0.001)

The transcript size of *Glyma.20G019300* (*GmNAP1*) is 4799 bp, and predicted protein length is 1388 aa (Fig. 4a). The predicted amino acid sequence of *Glyma.20G019300* reveals that it shares 77% identity with *AtNAP1*, which encodes a NCK-associated protein 1 in Arabidopsis (Fig. S2). NCK-associated protein 1 is a component of the WAVE complex, which includes Sra1/pir121/CYFIP1, Nap1/Nap125, Abi-1/Abi-2, Brick1 (Brk1)/HSPC3000, and SCAR/WAVE, and constitutes a large superfamily in plants. NAP1 has been identified in the genomes of *G. max* (Campbell et al. 2016), *M. truncatula* (Miyahara et al. 2010), *O. sativa* (Zhou et al. 2016), and *Arabidopsis* (Hulskamp et al. 1994). A previous study showed that the homolog of *NAP1* in soybean was *Glyma.20g019300*, which controls trichome development (Campbell et al. 2016). Amino acid sequences were aligned using the ClustalW multiple sequence alignment program, and a phylogenetic tree was generated using MEGA7 (Fig. S2). The results suggest that these proteins exist extensively in monocotyledons and dicotyledons and have a conserved function.

Syntenic conserved block analysis revealed that *GmNAP1* and *Glyma.07G221000* are more likely duplicated genes in soybean genome (Fig. 4a, b). The predicted size of transcript and amino acids of *Glyma.07G221000* are 1569 bp and 523 aa, which are 3234 bp and 865 amino acids shorter than those of *GmNAP1*, respectively (Fig. 4g). The synonymous nucleotide substitution rate (Ks) of the genes in the syntenic block suggested that *GmNAP1* and *Glyma.07G221000* were duplicated about 9.89 Myr ago, overlapping with the second genome duplication event (13 Myr ago) (Schmutz et al. 2010). The *NAP1* homologs in the soybean genome appeared to duplicate during the second genome duplication event, and sequencing comparison of *GmNAP1* and *Glyma.07G221000* indicated that *Glyma.07G221000* subsequently underwent a deletion event and produced a non-function protein (Fig. 4g, h). The expression of *GmNAP1* were higher in 5 out of 7 tissues than *Glyma.07G221000* (Fig. 4i). The results indicated the soybean genome now contains a single functional *NAP1* homolog, *GmNAP1*.

### *GmNAP1* involved in actin filament processes by regulating F-actin organization in trichome

**Fig. 5:**
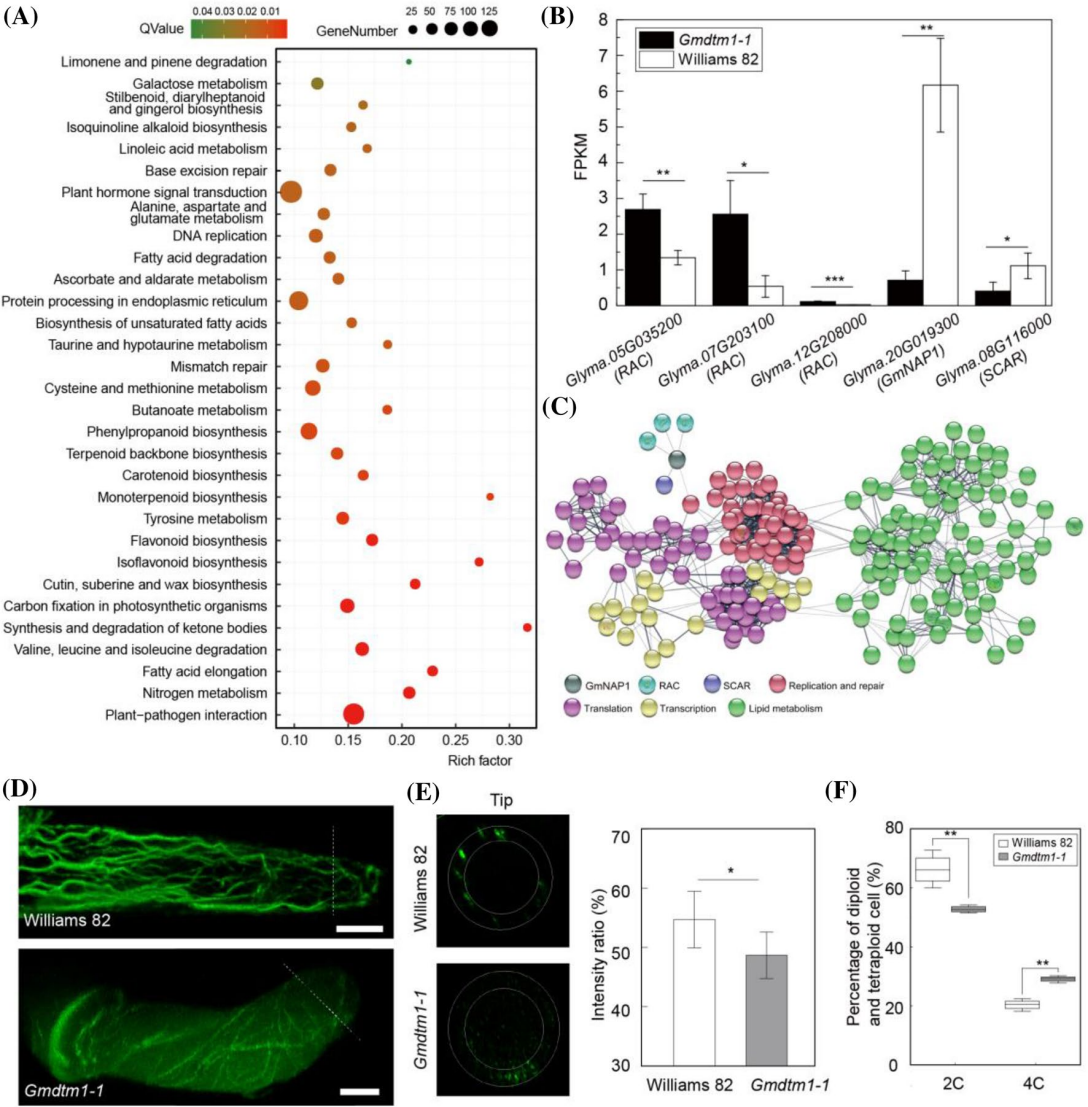
**a** Thirty-one important KEGG pathways enriched by DEGs between *Gmdtm1-1* and Williams 82. **b** Expression levels of actin filaments assembly genes. **c** Protein interaction network predicted by STRING (https://string-db.org) using DEGs. Replication and repair, translation, transcription, and lipid metabolism pathways are distinguished in different colors. **d** F-actin cytoskeletons of Williams 82 and *Gmdtm1-1*. Bars = 10 μm. **e** The integrated fluorescence intensity of transverse sections with 6 repeats taken at the top of the trichome. The values are the mean ratio ± standard deviation from 6 trichomes. **f** The percentage of diploid and tetraploid cells in *Gmdtm1-1* and Williams 82. The values are the mean ratio ± standard deviation with 4 biological repeats. Asterisks indicate significant differences as determined by Student’s *t*-test (*p < 0.05; **p < 0.01; ***p < 0.001)

To elucidate the pathway leading to the abnormal development of epidermal trichomes and pavement cells in *Gmdtm1-1*, we performed RNA-seq of *Gmdtm1-1* and Williams 82 using Illumina sequencing technology at the V2 growth stage when the second trifoliolate leaf was fully opened. The number of clean reads obtained from the raw reads ranged from 13,581,461,245 to 16,768,218,200 bp in the six samples (Table S2). About 85.01% of reference genes were detected. A total of 3040 genes (including 3834 transcripts) were up-regulated and 2061 genes (including 2858 transcripts) were down-regulated in *Gmdtm1-1* compared with Williams 82. The qRT-PCR results were consistent with the data derived from RNA-seq, demonstrating the reliability of our RNA-seq results (Fig. S1a, b). All DEGs (Differential Expression Genes) were mapped to the KEGG (https://www.kegg.jp) and 121 KEGG pathways were involved. Moreover, 31 pathways were identified with significant enrichment of DEGs (Fig. 5a).

The actin cytoskeleton of plant plays an important role in cell development, cell morphogenesis, and the establishment and maintenance of cell polarity. RACs, WAVE complex and Arp2/3 complex participate in the synthesis of the actin filament together (Yalovsky et al. 2008). We identified 20, 27 and 19 genes relating RACs, WAVE complex and ARP2/3 complex respectively (Table S5). According to the result of transcriptome analysis, 3 DEGs (*Glyma.05g035200*, *Glyma.07g203100*, *Glyma.12g208000*) which encode RAC protein were significantly up regulated compared with wild type (Fig. 5b, Table S5). Two DEGs (*Glyma.20g019300*, *Glyma.08g116000*) which belong to the SCAR/WAVE complex were significantly down regulated compared with wild type (Fig. 5b, Table S5). No DEGs of Arp2/3 complex were found (Table S5). Network analysis indicated that GmNAP1 protein interacted with RAC, SCAR and “replication and repair proteins (Fig. 5c). Compared with Williams 82, the tip of *Gmdtm1-1* trichomes has less abundant F-actin (Fig. 5d). The intensity ratio also significantly reduced in the tip of *Gmdtm1-1* trichome (Fig. 5e). Therefore, the distorted trichome of *Gmdtm1-1* is related with the reduced F-actin in the trichome tip.

### *GmNAP1* might affect the genetic information processing to regulate cell size of pavement cell

The transcriptome analysis identified 150 DEGs, including 136 down-regulated genes and four up-regulated genes, involved in pathways associated with “replication and repair,” “translation,” and “transcription,” which included 12 sub-pathways (Fig. S3b). Among the 150 DEGs between *Gmdtm1-1* and Williams 82, 108 DEGs (94 down-regulated and 14 up-regulated) were related with ‘replication and repair’ pathways (Table S3a). Of these, 27 DEGs (3 up-regulated and 24 down-regulated) were enriched in the “DNA replication” pathway (Fig. S3b, Table S3a). Genes associated with the DNA replication pathway, such as DNA polymerase α-primase complex, δ complex, ε complex, MCM complex, clamp loader complex, and helicase, were all down regulated (Table S3a). We also found 27 DEGs enriched in the “mismatch repair” pathway. The GO enrichment analysis also indicated that “DNA replication” and “transcription” were significantly enriched (Fig. S4). These genes, such as *MutL*, *MutS*, *RFC*, *Exonuclease*, *DNA polymerase δ*, and *DNA ligase I*, were down regulated (Fig. S3a), which was probably related to the pleotropic phenotypes to *Gmdtm1-1* and *Gmdtm1-2*.

The DNA contents of the mature pavement cells of wild-type and mutant were measured by flow cytometry to evaluate the effects of *GmNAP1* during genetic information processing. The ratio of diploid cells of *Gmdtm1-1* (52.75 ± 1.17 percentage of total cells) was significantly less than that of Williams 82 (66.18 ± 5.31 percentage of total cells). By contrast, the percentage of tetraploid cells of *Gmdtm1-1* (29.08 ± 1.04 percentage of total cells) increased significantly compared with Williams 82 (20.38 ± 1.78 percentage of total cells) (Figs. 5f, S3c). Increased DNA content or polyploidization is usually associated with increased cell size (Frawley and Orr-Weaver 2015; Orr-Weaver 2015). Therefore, the result suggests that the increased ratio of polyploidy cell of *Gmdtm1-1* might lead to the enlarge pavement cell.

### *Gmdtm1 *also affected plant height and yield

**Fig. 6:**
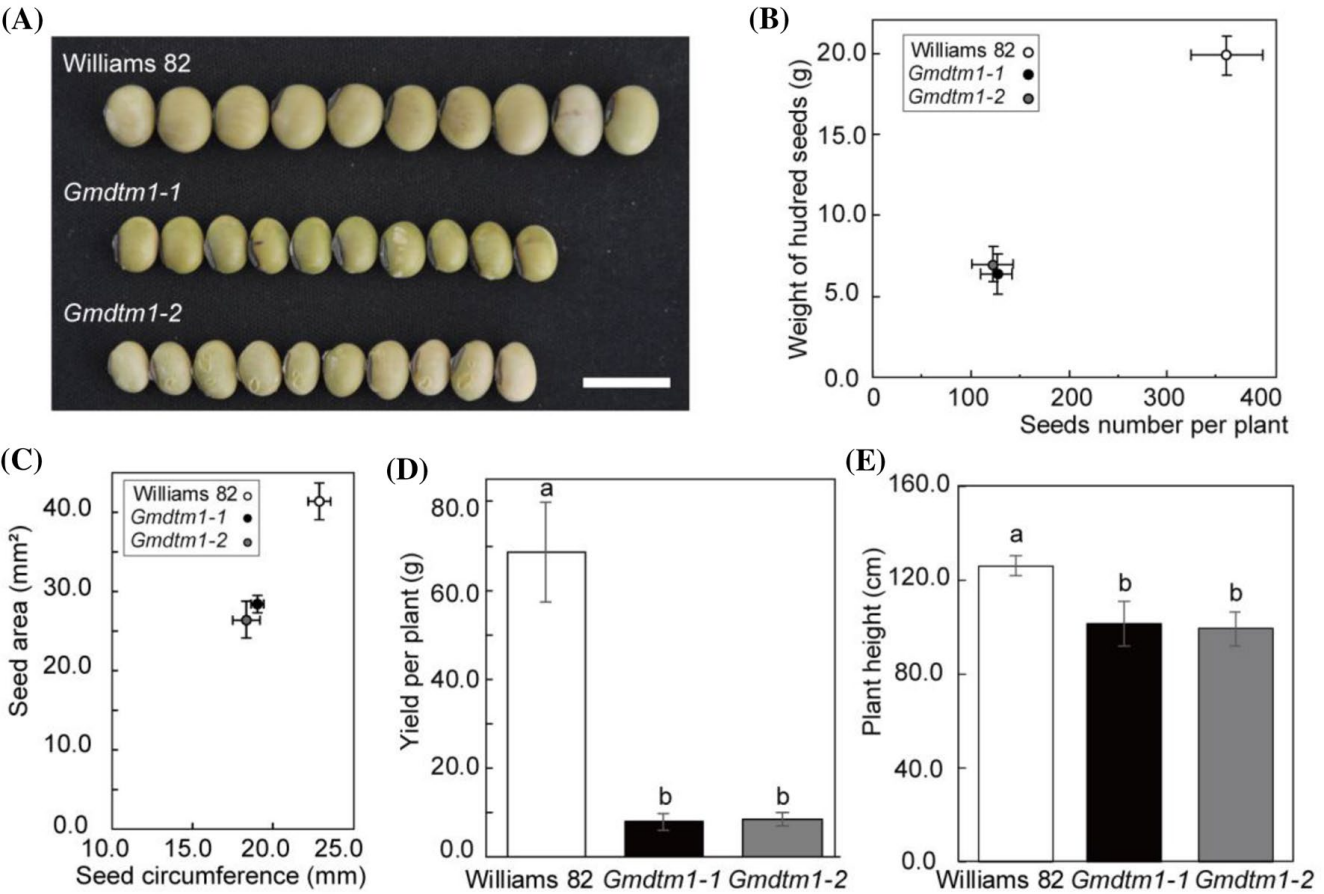
Yield indices of Williams 82, *Gmdtm1-1*, and *Gmdtm1-2.***a** Seed phenotype of Williams 82, *Gmdtm1-1*, and *Gmdtm1-2*. Bars = 1 cm. **b** Weight of 100 seeds and seeds number per plant of Williams 82, *Gmdtm1-1*, and *Gmdtm1-2*. **c** Seed area and seed perimeter of Williams 82, *Gmdtm1-1*, and *Gmdtm1-2*. **d** Yield per plant of Williams 82, *Gmdtm1-1*, and *Gmdtm1-2*. Data are presented as mean ± SD. **e** Plant height of Williams 82, *Gmdtm1-1*, and *Gmdtm1-2*. Data are presented as mean ± SD

*Gmdtm1* also showed the defects in plant growth and yield beside abnormal trichome development. *Gmdtm1-1* and *1-2* had reduced height and smaller seeds. The mean seed area in *Gmdtm1-1* and *1-2* was 28.21 ± 1.09 and 26.28 ± 2.33 mm^2^, respectively, which was decreased by 31.51% and 36.20% compared with Williams 82 (41.19 ± 2.31 mm^2^) (*p* < 0.05) (Fig. 6a, c). The seed circumference in the two mutants was 18.99 ± 0.39 and 18.45 ± 0.85 mm, which was decreased by 17.11% and 19.46% compared with Williams 82 (22.91 ± 0.70 mm) (Fig. 6a, c). The mean weight of 100 seeds in the two alleles was 6.34 ± 1.24 and 7.00 ± 1.12 g, respectively, which was significantly lower than Williams 82 (19.91 ± 1.20 g) (*p* < 0.05) (Fig. 6b). The plant yield in the two mutants was 7.94 ± 1.88 and 8.56 ± 1.43 g, respectively, which was significantly lower than Williams 82 (68.64 ± 11.12 g) (*p* < 0.05) (Fig. 6d). The plant height of *Gmdtm1-1* and *1-2* was 101.40 ± 9.40 and 99.20 ± 7.26 cm, respectively, at the R8 stage, which was decreased by 20% and 21% compared with that of ‘Williams’ 82 (126.08 ± 4.36 cm) (*p* < 0.05) (Fig. 6e). The various growth and development phenotypes of *Gmdtm1* reveals that *GmNAP1* is required for soybean growth and agronomic traits.

## Discussion

The SCAR/WAVE complex is involved in many processes contributing to important crop traits, such as stomatal dynamics and water use efficiency, infection thread formation during root nodulation, and control of cellular growth that impacts organ architecture and the adhesive properties of cells in the context of a tissue (Deeks et al. 2004; El-Assal Sel et al. 2004; Fu et al. 2013; Li et al. 2004; Ning et al. 2016; Zhou et al. 2016). The component of SCAR/WAVE complex, NAP (Nck-associated protein), has been reported to regulate actin-based cell morphogenesis and multiple developmental processes in *Arabidopsis* (Brembu et al. 2004; Deeks et al. 2004; El-Assal Sel et al. 2004; Fu et al. 2013; Li et al. 2004; Ning et al. 2016; Zhou et al. 2016). In rice, *less pronounced lobe epidermal cell3-1* (*lpl3-1*), encoding NCK-associated protein 1, developed a smooth surface, with fewer serrated pavement cell (PC) lobes, and decreased papillae (Zhou et al. 2016). *DS8* (*Drought Sensitive 8*) gene, a NAP1-like protein in rice, recently was reported to affect drought sensitivity by involvement leaf epidermal development and stomatal closure (Huang et al. 2019). In soybean, Campbell et al. (2016) identified a 26.6 megabase interval on chromosome 20 that co-segregated with the gnarled trichrome phenotype in a fast neutron mutant population. This chromosome 20 interval included a small structural variant within the coding region of a soybean NAP1 locus. A wild-type soybean NAP1 transgene functionally complemented an Arabidopsis *nap1* mutant. In this study, two EMS induced soybean trichrome mutants (*Gmdtm1-1* and *1-2*), were isolated and mapped to *Glyma.20G019300* gene independently by map-based cloning. The soybean transgenic complementation experiment clearly proved the funtion of *GmNAP1*. This work not only confirmed the previous results, but also dispelled doubts of its function because of tortuous genetic background of previous mutant. The WAVE/SCAR complex and ARP2/3 complex are important protein complexes belonging to the ROP small GTPase signal transduction pathway (Vernoud et al. 2003; Yanagisawa et al. 2013; Zhang et al. 2008), which promote actin polymerization by enhancing F-actin nucleation and side-binding activities that result in the initiation of fine actin filaments (Hulskamp 2004). In *Gmdtm1-1* mutant, 5 DEGs relating to WAVE complex were also found in this study (Tab. S5). The further characterization functions of these genes will help to resolve the contribution of SCAR/WAVE complex to soybean agronomic traits.

Epidermal pavement cells of most dicot flowering plant species have lobed morphologies (Smith and Oppenheimer 2005), and the actin filament plays a critical role in the spatial regulation of pavement cell growth (Pratap Sahi et al. 2017). Lobe initiation and outgrowth of the pavement cell appear to require cortical fine actin microfilaments localized to sites lacking well-ordered cortical microfilaments (Armour et al. 2015; Frank and Smith 2002; Fu et al. 2005). Actin filaments can be assembled both outside and inside the nucleus and may be involved in chromatin remodeling and transcriptional control (Olave et al. 2002). Actin filaments assembled outside the nucleus support the overall shape of the cell and aid in cellular organization, while actin filaments assembled inside the nucleus respond to multiple cellular perturbations, including heat shock, protein misfolding, integrin engagement, and serum stimulation (Belin et al. 2015). In our study, we found that the lobe and neck structures of pavement cells were nearly absent in the two mutants, *Gmdtm1-1* and *1-2*, especially the lobe. The reduced F-actin in the tip trichome (Fig. 5b) and increased ratio of polyploidy cell of *Gmdtm1-1* might closely related to the abnormal cells size and shape of both pavement cell and trichrome. Besides the pavement cell and trichrome, the plant height and seed size were also altered in mutant. We also noticed pathways, associated with “lipid metabolic process”, “fatty acid metabolic process” and “fatty acid biosynthetic process”, were significantly enriched in the GO enrichment analysis (Fig. S3a, Table S4). Further analysis of more factors different pathways related to *GmNAP1* will help to improve soybean varieties in the future.

## Materials and methods

### Plant materials

Seeds of soybean (*Glycine max*) cultivars Williams 82 and ‘Hedou 12’ were obtained from Chinese Academy of Agricultural Sciences and Jining Academy of Agricultural Sciences, respectively. *Glycine max distorted trichome mutant 1–1* and *1–2* (*Gmdtm1-1* and *Gmdtm1-2*) were identified from an ethyl methylsulfonate (EMS) mutagenized population of Williams 82 (Feng et al. 2019). The plant heights were measured at R8 stage; the seed number per plant, weight of hundred seeds, seed area and seed perimeter was measured after harvesting.

### Genetic mapping and bulked segregant analysis (BSA)

F2 plants derived from a cross between the mutant and ‘Hedou 12’ were used for mapping. Plants with distorted trichome phenotype were selected for preliminary mapping with about 165 InDel (insertion or deletion) markers between ‘Hedou 12’ and Williams 82 (Song et al. 2015). Fine-mapping oligos were developed using data from the whole-genome re-sequencing of ‘Hedou 12’ (Song et al. 2015).

DNA from individuals with the shorter trichome phenotype and individuals with wild-type phenotype was bulked into mutant and wild-type pools in an equal ratio. Paired-end sequencing libraries with an insert size of approximately 350 bp were sequenced on an Illumina HiSeq X Ten sequencer (Illumina, USA) at Novogene Biotech Company (Beijing, China). Bulked segregant analysis was performed as our previously work (Feng et al. 2019). Sequences were deposited at the National Center for Biotechnology Information (NCBI) with the accession number SRP149317.

### Phylogenetic analysis

The amino acid sequence of *GmNAP1* was used to identify homologous genes of *GmNAP1* in Phytozome (https://phytozome.jgi.doe.gov). A neighbor-joining tree was generated with the Poisson correction method using MEGA 7.0 software (Kumar et al. 2016). Bootstrap replication (1000 replications) was used to determine statistical support for the nodes in the phylogenetic tree. Microsynteny analysis was performed using MCSanX (Wang et al. 2012). Gene structure was analyzed as previous work (Dai et al. 2018).

### RNA-seq analysis and qPCR validation

Total RNA was isolated from young leaves using TRIzol, following the manufacturer’s methods (Invitrogen, Carlsbad, USA). Paired-end sequencing libraries with an insert size of approximately 350 bp were sequenced on an Illumina Hiseq X Ten platform at Novogene Biotech Company (Beijing, China). Sequences were deposited at the National Center for Biotechnology Information (NCBI) with the accession number SRP149402. Gene expression (FPKM, fragments per kilobase of transcript per million fragments mapped) levels were estimated using the Cufflinks software (version v 2.1.1) (Trapnell et al. 2012), and differentially expressed genes (DEGs) were selected by using the criteria *q* < 0.05 and |log_2_ (fold change)| ≥ 1. Reverse-transcription PCR was performed using a PrimeScript RT-PCR Kit (Takara, RR014) following the manufacturer’s methods. The samples used for qPCR were the same as the RNA-seq, which has three independent biological replicates, and the genes relative expression level were calculated using the 2^−∆∆Ct^ method after normalization to *Cons4* (*Glyma.12G020500*) (Libault et al. 2008).

### Scanning electron microscopy (SEM)

Mature leaves were cut into 1 cm squares, and fixed in 2.5% glutaraldehyde solution for SEM analysis (Zhou et al. 2016). The SEM images were acquired using HITACHI S-3400 and JEOL JSM-IT500. Pavement cell area and perimeter length, and trichome length were measured using ImageJ software (Schneider et al. 2012). Measurements of trichome density with three biological repeats were performed using images with 2 mm^2^ (2 × 1 mm). Trichome lengths with three biological repeats were measured for 10 trichome cells from each plant. Pavement cell areas and perimeter lengths were measured with three biological repeats using 20 epidermal cells from each plant.

### Actin cytoskeleton and Flow cytometry analysis

The actin staining performed as previously described (Zhou et al. 2016). The trichome of Williams 82 and *Gmdtm1-1* at VC stage were stained with iFluor 488 phalloidin, and the fluorescence images were projections of confocal sections (C2, Nikon). The integrated fluorescence intensity of transverse sections with 6 repeats taken at the top of the trichome using NIS Elements software (version 4.6). The core fluorescence was a wide ring around the perimeter from the trichome surface which occupied half area of transverse section.

The flow cytometry analysis was followed by the previous studies (Dolezel 1991; Dolezel and Bartos 2005). All samples were analyzed by flow cytometry (LSRFortessa, BD), FACSDiva software (version 6.1.3) and FlowJo software (version 10.6.1).

### Statistics analysis

All experiments were carried out using at least three biological repeats for each treatment and all statistical analyses were performed with R software (version 3.6.2). Pairwise-comparison was performed by Student’s *t*-test. Asterisks indicate significant differences as determined (*p < 0.05; **p < 0.01; ***p < 0.001). Multiple comparison tests were performed with multcomp R-package. Significance level was set at *p* < 0.05.

## Electronic supplementary material

Below is the link to the electronic supplementary material.

Supplementary file1 (DOCX 1039 kb)
