# Supplementary material for: GmNAP1 is essential for trichome and leaf epidermal cell development in soybean
Source: Plant Mol Biol. 2020 May 15;103(6):609–21. doi: 10.1007/s11103-020-01013-y (PMC7385028; doi:10.1007/s11103-020-01013-y)
Supplement: Supplementary file 1 — Supplementary file1 (DOCX 1039 kb) [file 11103_2020_1013_MOESM1_ESM.docx]

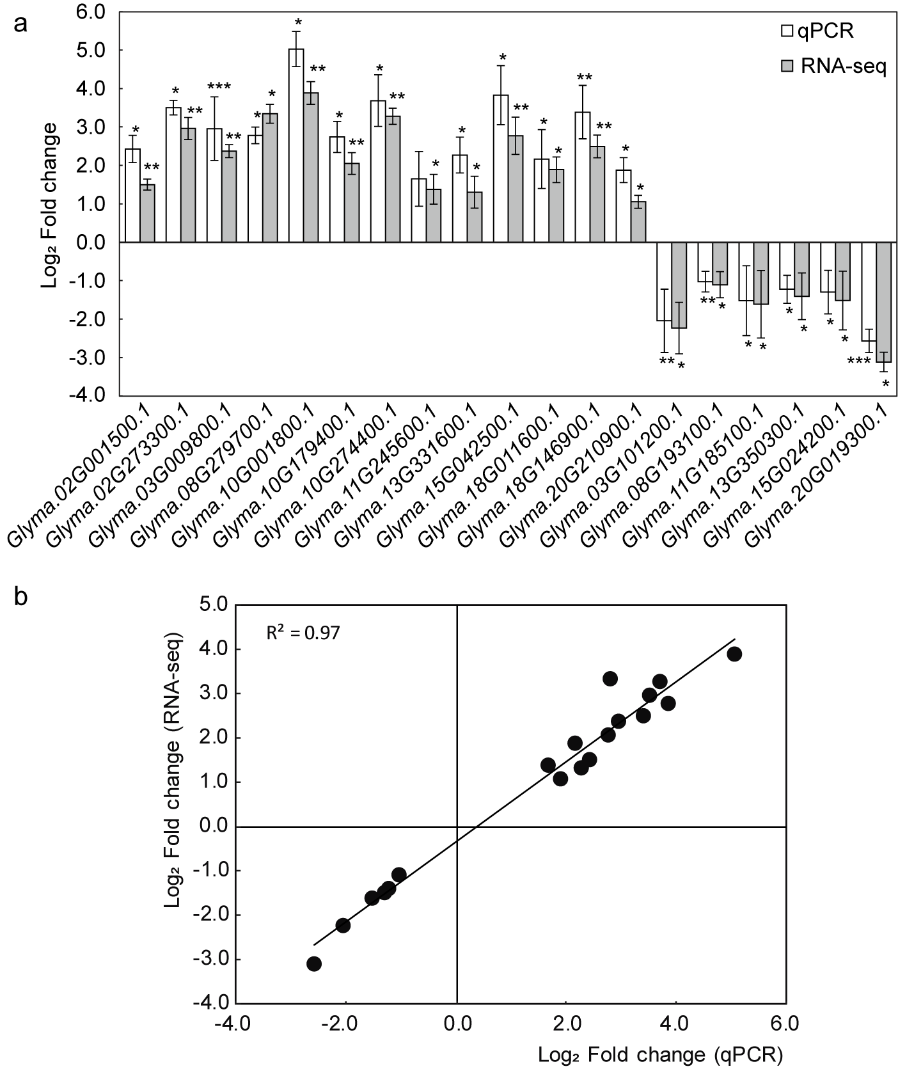


**Fig. S1** Verification of RNA-seq results using qRT-PCR. **a** Nineteen genes with *q*-value < 0.05 were selected from the RNA-seq data and their expression levels were assessed by qRT-PCR. Mean fold changes in expression of *Gmdtm1-1* and *Gmdtm1* obtained by qRT-PCR of three independent biological replicates were shown. Bars represent standard errors (SEs). Asterisks indicate significant differences as determined by Student’s *t*-test (**p*<0.05; ***p*<0.01; ****p*<0.001). **b** High correlation (R^2^ > 0.97) between expression changes of 19 genes obtained from qPCR and RNA-seq.


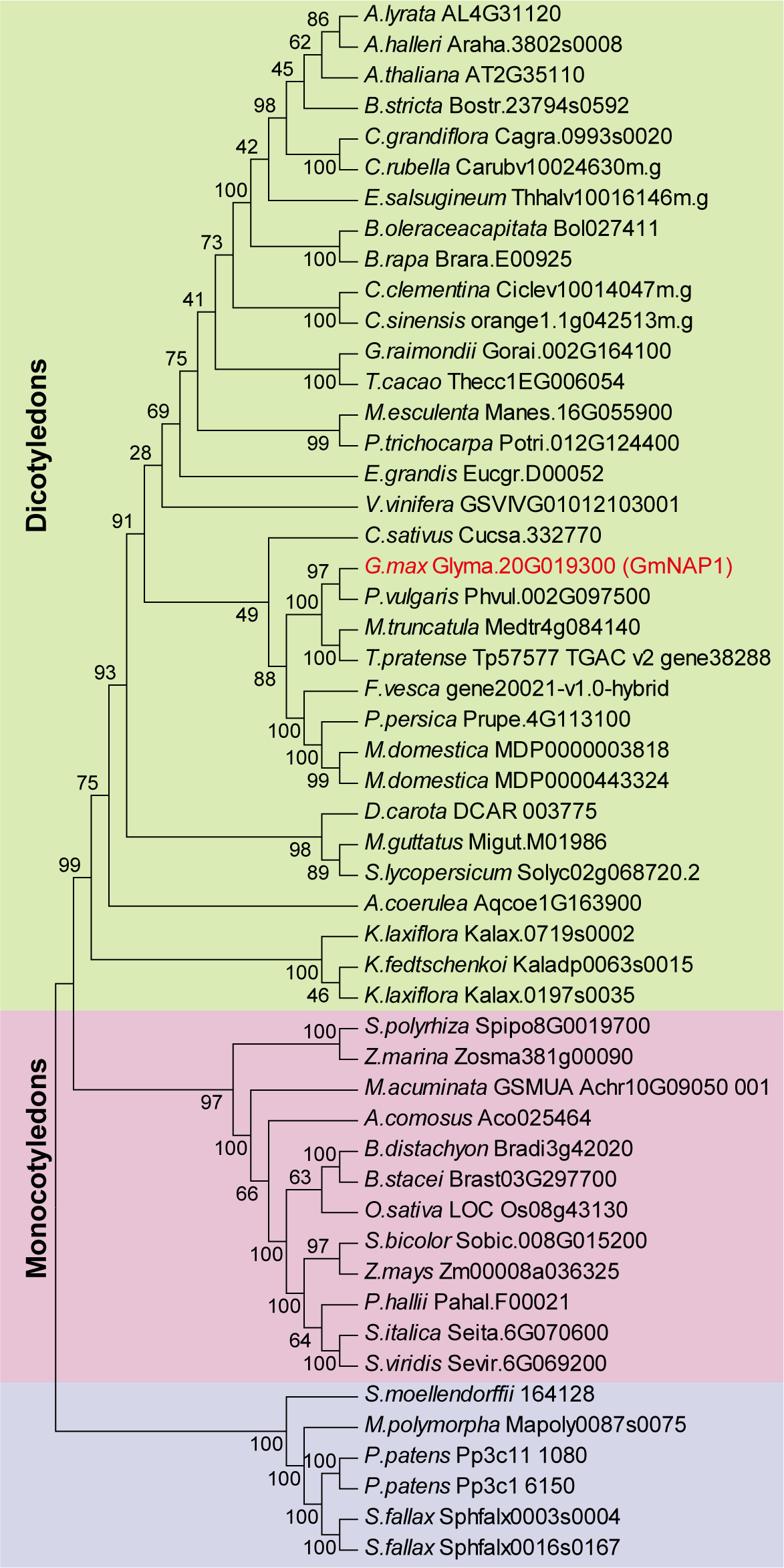


**Fig. S2** *GmNAP1* encodes a NCK-associated protein 1. Neighbor-joining phylogenetic tree and protein motifs of *GmNAP1* and other *NAP1* homologues in 51 plant species (species’ name and gene accession number indicating in the tree). Numbers on branches indicate percentage bootstrap support from 1000 replicates.


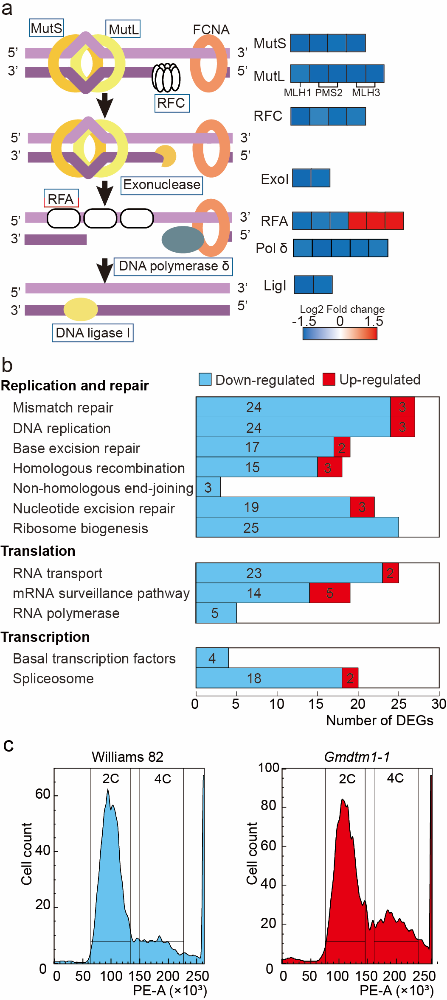


**Fig. S3**. Expression levels of DEGs associated with the “mismatch repair” pathway. **a** Expression levels of DEGs associated with the “mismatch repair” pathway. **b** Expression levels of DEGs in the pathway of “replication and repair”, “translation”, and “transcription” pathways. Numbers on the bars refer to the number of DEGs. **c** The flow cytometry spectrograms of the Williams 82 and *Gmdtm1-1*. PE-A (phycoerythrin-area) represents the relative fluorescence intensity. 2C and 4C represents diploid and tetraploid cells, respectively.

**
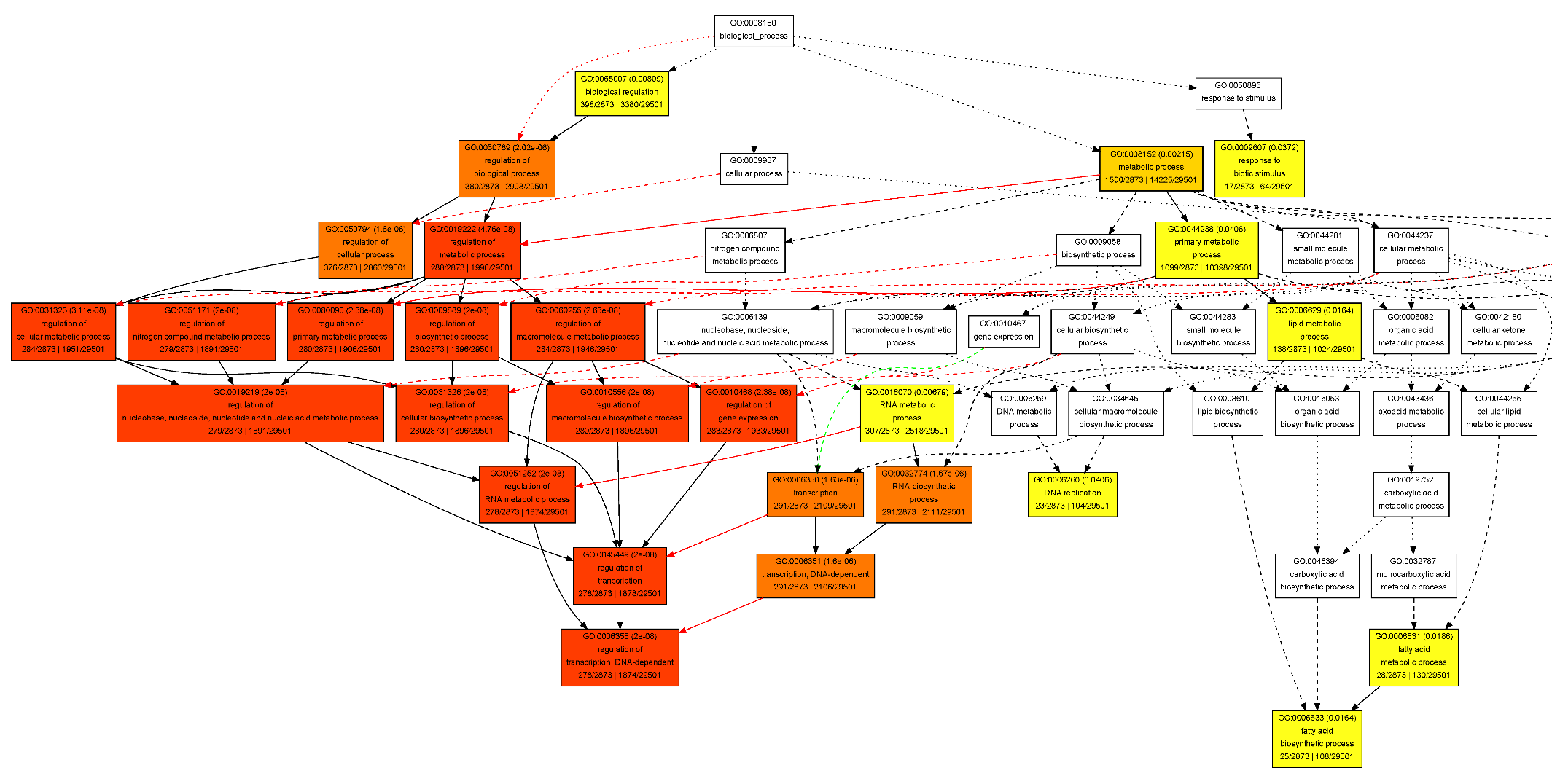
**

**Fig. S4 GO enrichment analysis of DEGs in the *Gmdtm1* mutant.**

The Go enrichment analysis of DEGs between Williams 82 and *Gmdtm1* mutant was performed by using AgriGO analysis toolkit (http://bioinfo.cau.edu.cn/agriGO). The parameters of each box indicated the GO term number, the p-value in parenthesis, and GO term respectively. The significance in each gene ontology category is indicated from white (not significant) to red (highly significant).

**Tab. S1** Seven candidate genes between markers OL6787 and OL6756.

| **Gene name^a^** | **Description^a^** |
| --- | --- |
| *Glyma.20G018900* | Cytochrome P450, family 87, subfamily A, polypeptide 6 |
| *Glyma.20G019000* | Exostosin family protein |
| *Glyma.20G019100* | Sulfiredoxin |
| *Glyma.20G019200* | DNA-directed RNA polymerase II subunit RPB3 |
| *Glyma.20G019300* | NCK-associated protein 1 (NCKAP1, NAP125) |
| *Glyma.20G019400* | TCP-1/cpn60 chaperonin family |
| *Glyma.20G019500* | Thiamine-phosphate diphosphorylase |

^a^ From https://phytozome.jgi.doe.gov

**Tab. S2** Summary of raw reads and reference statistics for the RNAseq of *Gmdtm1-1* and Williams 82.

| **Sample Name** | **Data (bp)** | **CycleQ20 (%)** | **GC (%)** | **Unique Mapped Reads (%)** | **Known Isoform Number (%)** |
| --- | --- | --- | --- | --- | --- |
| *Gmdtm1-1* | 13,581,461,245 | 96.41 | 43.57 | 87.99 | 78.87 |
| *Gmdtm1-1* | 15,276,981,690 | 96.4 | 45.84 | 84.74 | 76.84 |
| *Gmdtm1-1* | 14,829,582,027 | 96.42 | 45.7 | 84.09 | 76.57 |
| Williams 82 | 16,485,152,688 | 96.38 | 43.93 | 87.99 | 77.71 |
| Williams 82 | 16,768,218,200 | 96.62 | 45.65 | 81.87 | 78.96 |
| Williams 82 | 14,376,654,954 | 96.67 | 44.87 | 88.54 | 77.89 |

Data are total nucleotides after filtered. GC (%) is the proportion of guanidine and cytosine nucleotides among the total nucleotides. CycleQ20 (%) is the proportion of nucleotides with a quality value larger than 20. Unique Mapped reads represents the proportion of reads and comparison with reference genome alignment to a single position. Known isoform number is the proportion of the gene detected from the sequence in each sample.

**Tab.** **S3** The DEGs associated with “replication and repair”, “translation” and “transcription” pathway.

(The up regulated genes are colored with red, and the down regulated genes are colored with green.)

| **Pathway Class** | **Pathway** | **Gene name** | **log_2_FC** | **FDR** | **Description** |
| --- | --- | --- | --- | --- | --- |
| Replication and repair | Mismatch repair | *Glyma.01G158400* | -1.71 | 0.01 | MUTL protein homolog 3 |
| Replication and repair | Mismatch repair | *Glyma.02G155200* | -1.49 | 0.02 | DNA mismatch repair protein, putative |
| Replication and repair | Mismatch repair | *Glyma.04G210700* | -1.55 | 0.01 | DNA binding; nucleotide binding; nucleic acid binding; DNA-directed DNA polymerases; DNA-directed DNA polymerases |
| Replication and repair | Mismatch repair | *Glyma.04G254900* | -1.34 | 0.01 | MUTL-homologue 1 |
| Replication and repair | Mismatch repair | *Glyma.05G020600* | -1.43 | 0.02 | RPA70-kDa subunit B |
| Replication and repair | Mismatch repair | *Glyma.06G116000* | -1.48 | 0.01 | ATPase family associated with various cellular activities (AAA) |
| Replication and repair | Mismatch repair | *Glyma.06G155300* | -1.39 | 0.02 | DNA binding; nucleotide binding; nucleic acid binding; DNA-directed DNA polymerases; DNA-directed DNA polymerases |
| Replication and repair | Mismatch repair | *Glyma.06G308700* | -1.19 | 0.05 | replication factor C1 |
| Replication and repair | Mismatch repair | *Glyma.07G087700* | -1.59 | 0.01 | MUTS homolog 7 |
| Replication and repair | Mismatch repair | *Glyma.08G120900* | -1.37 | 0.02 | 5\'-3\' exonuclease family protein |
| Replication and repair | Mismatch repair | *Glyma.09G189100* | -1.75 | 0.00 | MUTS homolog 7 |
| Replication and repair | Mismatch repair | *Glyma.10G278800* | -1.26 | 0.04 | Nucleic acid-binding, OB-fold-like protein |
| Replication and repair | Mismatch repair | *Glyma.11G193100* | -1.40 | 0.05 | DNA ligase 1 |
| Replication and repair | Mismatch repair | *Glyma.12G080900* | -1.45 | 0.02 | DNA ligase 1 |
| Replication and repair | Mismatch repair | *Glyma.17G130800* | -1.26 | 0.03 | replication factor C 2 |
| Replication and repair | Mismatch repair | *Glyma.19G147500* | -1.28 | 0.03 | DNA polymerase delta small subunit |
| Replication and repair | Mismatch repair | *Glyma.19G216000* | -1.44 | 0.01 | MUTS homolog 6 |
| Replication and repair | Mismatch repair | *Glyma.20G111700* | -1.25 | 0.05 | Nucleic acid-binding, OB-fold-like protein |
| Replication and repair | Mismatch repair | *Glyma.03G121900* | 6.54 | 0.03 | Nucleic acid-binding, OB-fold-like protein |
| Replication and repair | Mismatch repair | *Glyma.04G181700* | 6.44 | 0.02 | Nucleic acid-binding, OB-fold-like protein |
| Replication and repair | Mismatch repair | *Glyma.13G029300* | 4.23 | 0.00 | RPA70-kDa subunit B |
| Replication and repair | Mismatch repair | *TCONS_00001375* | -1.71 | 0.00 | PREDICTED: DNA mismatch repair protein MLH3-like isoform X1 |
| Replication and repair | Mismatch repair | *TCONS_00005882* | -1.48 | 0.01 | PREDICTED: DNA mismatch repair protein PMS1-like |
| Replication and repair | Mismatch repair | *TCONS_00017342* | -1.41 | 0.02 | PREDICTED: DNA polymerase delta catalytic subunit isoform X3 |
| Replication and repair | Mismatch repair | *TCONS_00034584* | -1.88 | 0.01 | PREDICTED: exonuclease 1 |
| Replication and repair | Mismatch repair | *TCONS_00047617* | -1.83 | 0.00 | PREDICTED: DNA mismatch repair protein MSH7-like isoform X2 |
| Replication and repair | Mismatch repair | *TCONS_00065981* | -1.32 | 0.04 | DNA polymerase delta subunit 3 |
| Replication and repair | DNA replication | *Glyma.01G187400* | -1.70 | 0.01 | DNA-directed DNA polymerases |
| Replication and repair | DNA replication | *Glyma.04G210700* | -1.55 | 0.01 | DNA binding; nucleotide binding; nucleic acid binding; DNA-directed DNA polymerases; DNA-directed DNA polymerases |
| Replication and repair | DNA replication | *Glyma.05G020600* | -1.43 | 0.02 | RPA70-kDa subunit B |
| Replication and repair | DNA replication | *Glyma.06G116000* | -1.48 | 0.01 | ATPase family associated with various cellular activities (AAA) |
| Replication and repair | DNA replication | *Glyma.06G155300* | -1.39 | 0.02 | DNA binding; nucleotide binding; nucleic acid binding; DNA-directed DNA polymerases; DNA-directed DNA polymerases |
| Replication and repair | DNA replication | *Glyma.06G308700* | -1.19 | 0.05 | replication factor C1 |
| Replication and repair | DNA replication | *Glyma.06G310500* | -1.36 | 0.04 | DNA replication helicase, putative |
| Replication and repair | DNA replication | *Glyma.10G141800* | -1.22 | 0.03 | 5\'-3\' exonuclease family protein |
| Replication and repair | DNA replication | *Glyma.10G278800* | -1.26 | 0.04 | Nucleic acid-binding, OB-fold-like protein |
| Replication and repair | DNA replication | *Glyma.11G183100* | -1.47 | 0.01 | nuclear factor Y, subunit B11 |
| Replication and repair | DNA replication | *Glyma.11G193100* | -1.40 | 0.05 | DNA ligase 1 |
| Replication and repair | DNA replication | *Glyma.11G247900* | -1.70 | 0.01 | DNA polymerase alpha 2 |
| Replication and repair | DNA replication | *Glyma.12G080900* | -1.45 | 0.02 | DNA ligase 1 |
| Replication and repair | DNA replication | *Glyma.12G087000* | -1.74 | 0.01 | DNA polymerase epsilon catalytic subunit |
| Replication and repair | DNA replication | *Glyma.15G154900* | -1.28 | 0.04 | minichromosome maintenance (MCM2/3/5) family protein |
| Replication and repair | DNA replication | *Glyma.16G218700* | -1.37 | 0.01 | DNA polymerase epsilon subunit B2 |
| Replication and repair | DNA replication | *Glyma.17G130800* | -1.26 | 0.03 | replication factor C 2 |
| Replication and repair | DNA replication | *Glyma.18G009300* | -1.42 | 0.02 | DNA polymerase alpha 2 |
| Replication and repair | DNA replication | *Glyma.19G147500* | -1.28 | 0.03 | DNA polymerase delta small subunit |
| Replication and repair | DNA replication | *Glyma.19G216100* | -1.37 | 0.02 | Minichromosome maintenance (MCM2/3/5) family protein |
| Replication and repair | DNA replication | *Glyma.20G111700* | -1.25 | 0.05 | Nucleic acid-binding, OB-fold-like protein |
| Replication and repair | DNA replication | *TCONS_00017342* | -1.41 | 0.02 | PREDICTED: DNA polymerase delta catalytic subunit isoform X3 |
| Replication and repair | DNA replication | *TCONS_00056463* | -1.45 | 0.03 | PREDICTED: DNA polymerase alpha subunit B-like isoform X1 |
| Replication and repair | DNA replication | *TCONS_00065981* | -1.32 | 0.04 | DNA polymerase delta subunit 3 |
| Replication and repair | DNA replication | *Glyma.03G121900* | 6.54 | 0.03 | Nucleic acid-binding, OB-fold-like protein |
| Replication and repair | DNA replication | *Glyma.04G181700* | 6.44 | 0.02 | Nucleic acid-binding, OB-fold-like protein |
| Replication and repair | DNA replication | *Glyma.13G029300* | 4.23 | 0.00 | RPA70-kDa subunit B |
| Replication and repair | Base excision repair | *Glyma.02G123800* | -2.35 | 0.00 | DNA glycosylase superfamily protein |
| Replication and repair | Base excision repair | *Glyma.03G161300* | -2.31 | 0.00 | poly(ADP-ribose) polymerase 2 |
| Replication and repair | Base excision repair | *Glyma.04G210700* | -1.55 | 0.01 | DNA binding; nucleotide binding; nucleic acid binding; DNA-directed DNA polymerases; DNA-directed DNA polymerases |
| Replication and repair | Base excision repair | *Glyma.06G155300* | -1.39 | 0.02 | DNA binding; nucleotide binding; nucleic acid binding; DNA-directed DNA polymerases; DNA-directed DNA polymerases |
| Replication and repair | Base excision repair | *Glyma.08G263200* | -2.24 | 0.00 | DNA glycosylase superfamily protein |
| Replication and repair | Base excision repair | *Glyma.10G141800* | -1.22 | 0.03 | 5\'-3\' exonuclease family protein |
| Replication and repair | Base excision repair | *Glyma.11G183100* | -1.47 | 0.01 | nuclear factor Y, subunit B11 |
| Replication and repair | Base excision repair | *Glyma.11G193100* | -1.40 | 0.05 | DNA ligase 1 |
| Replication and repair | Base excision repair | *Glyma.12G080900* | -1.45 | 0.02 | DNA ligase 1 |
| Replication and repair | Base excision repair | *Glyma.12G087000* | -1.74 | 0.01 | DNA polymerase epsilon catalytic subunit |
| Replication and repair | Base excision repair | *Glyma.16G116300* | -1.42 | 0.01 | high mobility group B2 |
| Replication and repair | Base excision repair | *Glyma.16G218700* | -1.37 | 0.01 | DNA polymerase epsilon subunit B2 |
| Replication and repair | Base excision repair | *Glyma.19G147500* | -1.28 | 0.03 | DNA polymerase delta small subunit |
| Replication and repair | Base excision repair | *Glyma.19G162800* | -1.60 | 0.02 | poly(ADP-ribose) polymerase 2 |
| Replication and repair | Base excision repair | *TCONS_00017342* | -1.41 | 0.02 | PREDICTED: DNA polymerase delta catalytic subunit isoform X3 |
| Replication and repair | Base excision repair | *TCONS_00057461* | -1.46 | 0.03 | PREDICTED: uracil-DNA glycosylase, mitochondrial-like |
| Replication and repair | Base excision repair | *TCONS_00065981* | -1.32 | 0.04 | DNA polymerase delta subunit 3 |
| Replication and repair | Base excision repair | *Glyma.04G033700* | 1.73 | 0.00 | DNA glycosylase superfamily protein |
| Replication and repair | Base excision repair | *Glyma.08G232900* | 2.00 | 0.00 | DNA glycosylase superfamily protein |
| Replication and repair | Homologous recombination | *Glyma.01G244700* | -1.81 | 0.00 | homolog of RAD54 |
| Replication and repair | Homologous recombination | *Glyma.04G210700* | -1.55 | 0.01 | DNA binding; nucleotide binding; nucleic acid binding; DNA-directed DNA polymerases; DNA-directed DNA polymerases |
| Replication and repair | Homologous recombination | *Glyma.05G020600* | -1.43 | 0.02 | RPA70-kDa subunit B |
| Replication and repair | Homologous recombination | *Glyma.06G155300* | -1.39 | 0.02 | DNA binding; nucleotide binding; nucleic acid binding; DNA-directed DNA polymerases; DNA-directed DNA polymerases |
| Replication and repair | Homologous recombination | *Glyma.06G155600* | -1.90 | 0.04 | topoisomerase 3alpha |
| Replication and repair | Homologous recombination | *Glyma.08G188100* | -1.62 | 0.00 | DNA helicase (RECQl4A) |
| Replication and repair | Homologous recombination | *Glyma.10G278800* | -1.26 | 0.04 | Nucleic acid-binding, OB-fold-like protein |
| Replication and repair | Homologous recombination | *Glyma.13G010700* | -1.44 | 0.01 | BRCA2-like B |
| Replication and repair | Homologous recombination | *Glyma.14G055000* | -1.85 | 0.02 | DNA topoisomerase, type IA, core |
| Replication and repair | Homologous recombination | *Glyma.19G147500* | -1.28 | 0.03 | DNA polymerase delta small subunit |
| Replication and repair | Homologous recombination | *Glyma.20G111700* | -1.25 | 0.05 | Nucleic acid-binding, OB-fold-like protein |
| Replication and repair | Homologous recombination | *TCONS_00002132* | -1.74 | 0.00 | PREDICTED: protein CHROMATIN REMODELING 25 |
| Replication and repair | Homologous recombination | *TCONS_00017342* | -1.41 | 0.02 | PREDICTED: DNA polymerase delta catalytic subunit isoform X3 |
| Replication and repair | Homologous recombination | *TCONS_00065981* | -1.32 | 0.04 | DNA polymerase delta subunit 3 |
| Replication and repair | Homologous recombination | *TCONS_00075297* | -2.14 | 0.05 | PREDICTED: DNA repair protein RAD51 homolog 2 isoform X2 |
| Replication and repair | Homologous recombination | *Glyma.03G121900* | 6.54 | 0.03 | Nucleic acid-binding, OB-fold-like protein |
| Replication and repair | Homologous recombination | *Glyma.04G181700* | 6.44 | 0.02 | Nucleic acid-binding, OB-fold-like protein |
| Replication and repair | Homologous recombination | *Glyma.13G029300* | 4.23 | 0.00 | RPA70-kDa subunit B |
| Replication and repair | Non-homologous end-joining | *Glyma.10G141800* | -1.22 | 0.03 | 5\'-3\' exonuclease family protein |
| Replication and repair | Non-homologous end-joining | *TCONS_00027313* | -1.35 | 0.03 | PREDICTED: ATP-dependent DNA helicase 2 subunit KU80 |
| Replication and repair | Non-homologous end-joining | *TCONS_00086751* | -2.39 | 0.00 | PREDICTED: ATP-dependent DNA helicase 2 subunit KU80-like |
| Replication and repair | Nucleotide excision repair | *Glyma.01G077200* | -1.35 | 0.02 | 5\'-3\' exonuclease family protein |
| Replication and repair | Nucleotide excision repair | *Glyma.04G210700* | -1.55 | 0.01 | DNA binding; nucleotide binding; nucleic acid binding; DNA-directed DNA polymerases; DNA-directed DNA polymerases |
| Replication and repair | Nucleotide excision repair | *Glyma.05G020600* | -1.43 | 0.02 | RPA70-kDa subunit B |
| Replication and repair | Nucleotide excision repair | *Glyma.06G116000* | -1.48 | 0.01 | ATPase family associated with various cellular activities (AAA) |
| Replication and repair | Nucleotide excision repair | *Glyma.06G155300* | -1.39 | 0.02 | DNA binding; nucleotide binding; nucleic acid binding; DNA-directed DNA polymerases; DNA-directed DNA polymerases |
| Replication and repair | Nucleotide excision repair | *Glyma.06G308700* | -1.19 | 0.05 | replication factor C1 |
| Replication and repair | Nucleotide excision repair | *Glyma.08G096100* | -1.28 | 0.05 | cullin4 |
| Replication and repair | Nucleotide excision repair | *Glyma.10G278800* | -1.26 | 0.04 | Nucleic acid-binding, OB-fold-like protein |
| Replication and repair | Nucleotide excision repair | *Glyma.11G183100* | -1.47 | 0.01 | nuclear factor Y, subunit B11 |
| Replication and repair | Nucleotide excision repair | *Glyma.11G193100* | -1.40 | 0.05 | DNA ligase 1 |
| Replication and repair | Nucleotide excision repair | *Glyma.12G080900* | -1.45 | 0.02 | DNA ligase 1 |
| Replication and repair | Nucleotide excision repair | *Glyma.12G087000* | -1.74 | 0.01 | DNA polymerase epsilon catalytic subunit |
| Replication and repair | Nucleotide excision repair | *Glyma.14G214000* | -1.18 | 0.05 | DNA repair protein Rad4 family |
| Replication and repair | Nucleotide excision repair | *Glyma.16G218700* | -1.37 | 0.01 | DNA polymerase epsilon subunit B2 |
| Replication and repair | Nucleotide excision repair | *Glyma.17G130800* | -1.26 | 0.03 | replication factor C 2 |
| Replication and repair | Nucleotide excision repair | *Glyma.19G147500* | -1.28 | 0.03 | DNA polymerase delta small subunit |
| Replication and repair | Nucleotide excision repair | *Glyma.20G111700* | -1.25 | 0.05 | Nucleic acid-binding, OB-fold-like protein |
| Replication and repair | Nucleotide excision repair | *TCONS_00017342* | -1.41 | 0.02 | PREDICTED: DNA polymerase delta catalytic subunit isoform X3 |
| Replication and repair | Nucleotide excision repair | *TCONS_00065981* | -1.32 | 0.04 | DNA polymerase delta subunit 3 |
| Replication and repair | Nucleotide excision repair | *Glyma.03G121900* | 6.54 | 0.03 | Nucleic acid-binding, OB-fold-like protein |
| Replication and repair | Nucleotide excision repair | *Glyma.04G181700* | 6.44 | 0.02 | Nucleic acid-binding, OB-fold-like protein |
| Replication and repair | Nucleotide excision repair | *Glyma.13G029300* | 4.23 | 0.00 | RPA70-kDa subunit B |
| Translation | Ribosome biogenesis in eukaryotes | *Glyma.01G176400* | -1.46 | 0.01 |  |
| Translation | Ribosome biogenesis in eukaryotes | *Glyma.02G290200* | -1.57 | 0.01 | ARM repeat superfamily protein |
| Translation | Ribosome biogenesis in eukaryotes | *Glyma.03G186500* | -1.31 | 0.03 | transducin family protein / WD-40 repeat family protein |
| Translation | Ribosome biogenesis in eukaryotes | *Glyma.04G200700* | -1.86 | 0.00 | fibrillarin 2 |
| Translation | Ribosome biogenesis in eukaryotes | *Glyma.05G038000* | -1.68 | 0.00 | Transducin family protein / WD-40 repeat family protein |
| Translation | Ribosome biogenesis in eukaryotes | *Glyma.05G185000* | -1.48 | 0.01 | Domain of unknown function (DUF1726); Putative ATPase (DUF699) |
| Translation | Ribosome biogenesis in eukaryotes | *Glyma.07G032500* | -2.04 | 0.01 |  |
| Translation | Ribosome biogenesis in eukaryotes | *Glyma.07G035600* | -1.87 | 0.01 | ATPases; nucleotide binding; ATP binding; nucleoside-triphosphatases; transcription factor binding |
| Translation | Ribosome biogenesis in eukaryotes | *Glyma.08G143000* | -1.40 | 0.03 | Domain of unknown function (DUF1726); Putative ATPase (DUF699) |
| Translation | Ribosome biogenesis in eukaryotes | *Glyma.09G084600* | -1.50 | 0.01 | periodic tryptophan protein 2 |
| Translation | Ribosome biogenesis in eukaryotes | *Glyma.09G286500* | -1.62 | 0.02 | ribonuclease Ps |
| Translation | Ribosome biogenesis in eukaryotes | *Glyma.11G011700* | -1.28 | 0.04 | exportin 1A |
| Translation | Ribosome biogenesis in eukaryotes | *Glyma.11G068600* | -1.57 | 0.01 |  |
| Translation | Ribosome biogenesis in eukaryotes | *Glyma.11G237200* | -1.44 | 0.03 | fibrillarin 2 |
| Translation | Ribosome biogenesis in eukaryotes | *Glyma.12G049700* | -1.77 | 0.01 | P-loop containing nucleoside triphosphate hydrolases superfamily protein |
| Translation | Ribosome biogenesis in eukaryotes | *Glyma.14G024600* | -1.83 | 0.00 | ARM repeat superfamily protein |
| Translation | Ribosome biogenesis in eukaryotes | *Glyma.16G004800* | -1.72 | 0.00 | ATPases; nucleotide binding; ATP binding; nucleoside-triphosphatases; transcription factor binding |
| Translation | Ribosome biogenesis in eukaryotes | *Glyma.17G088200* | -1.76 | 0.03 |  |
| Translation | Ribosome biogenesis in eukaryotes | *Glyma.19G186600* | -1.68 | 0.01 | transducin family protein / WD-40 repeat family protein |
| Translation | Ribosome biogenesis in eukaryotes | *TCONS_00020655* | -1.38 | 0.02 | Transducin beta-like protein 3, partial |
| Translation | Ribosome biogenesis in eukaryotes | *TCONS_00045836* | -1.32 | 0.03 | Ribonucleases P/MRP protein subunit POP1 |
| Translation | Ribosome biogenesis in eukaryotes | *TCONS_00047003* | -1.94 | 0.00 | Nucleolar GTP-binding protein 1 |
| Translation | Ribosome biogenesis in eukaryotes | *TCONS_00047004* | -1.87 | 0.00 | Nucleolar GTP-binding protein 1 |
| Translation | Ribosome biogenesis in eukaryotes | *TCONS_00059412* | -1.94 | 0.01 | PREDICTED: ribosome biogenesis protein BMS1 homolog isoform X2 |
| Translation | Ribosome biogenesis in eukaryotes | *TCONS_00104775* | -2.44 | 0.01 | Os04g0548300, partial |
| Translation | RNA transport | *Glyma.02G305200* | -1.40 | 0.03 | Nucleoporin, Nup133/Nup155-like |
| Translation | RNA transport | *Glyma.05G001200* | -1.28 | 0.01 | Translation initiation factor IF2/IF5 |
| Translation | RNA transport | *Glyma.06G222100* | -1.76 | 0.00 | Protein of unknown function (DUF3414) |
| Translation | RNA transport | *Glyma.08G162300* | -1.21 | 0.05 | nuclear pore anchor |
| Translation | RNA transport | *Glyma.08G172000* | -1.28 | 0.04 | arginine/serine-rich 45 |
| Translation | RNA transport | *Glyma.09G286500* | -1.62 | 0.02 | ribonuclease Ps |
| Translation | RNA transport | *Glyma.11G011700* | -1.28 | 0.04 | exportin 1A |
| Translation | RNA transport | *Glyma.11G059700* | -1.23 | 0.04 | hydroxyproline-rich glycoprotein family protein |
| Translation | RNA transport | *Glyma.12G024600* | -1.70 | 0.00 | Protein of unknown function (DUF3414) |
| Translation | RNA transport | *Glyma.13G142200* | -1.44 | 0.02 | SUPPRESSOR OF AUXIN RESISTANCE1 |
| Translation | RNA transport | *Glyma.14G008200* | -1.30 | 0.04 | Nucleoporin, Nup133/Nup155-like |
| Translation | RNA transport | *Glyma.15G246500* | -2.49 | 0.00 | embryo defective 3012 |
| Translation | RNA transport | *Glyma.18G028200* | -2.23 | 0.02 | nuclear pore complex protein-related |
| Translation | RNA transport | *Glyma.18G040400* | -1.21 | 0.03 | ALWAYS EARLY 4 |
| Translation | RNA transport | *Glyma.U016500* | -1.34 | 0.01 |  |
| Translation | RNA transport | *TCONS_00045836* | -1.32 | 0.03 | Ribonucleases P/MRP protein subunit POP1 |
| Translation | RNA transport | *TCONS_00059147* | -1.62 | 0.00 | PREDICTED: nucleoporin NUP188 homolog |
| Translation | RNA transport | *TCONS_00059148* | -1.55 | 0.00 | PREDICTED: nucleoporin NUP188 homolog |
| Translation | RNA transport | *TCONS_00071567* | -2.05 | 0.01 | PHAX RNA-binding domain protein |
| Translation | RNA transport | *TCONS_00088933* | -3.46 | 0.02 | Trimethylguanosine synthase |
| Translation | RNA transport | *TCONS_00089395* | -2.11 | 0.01 | PREDICTED: nuclear pore complex protein NUP88-like |
| Translation | RNA transport | *TCONS_00089396* | -3.51 | 0.00 | PREDICTED: nuclear pore complex protein NUP88-like |
| Translation | RNA transport | *TCONS_00090968* | -8.20 | 0.00 | Eukaryotic translation initiation factor 3 subunit B |
| Translation | RNA transport | *Glyma.02G291500* | 1.56 | 0.02 | Translation initiation factor SUI1 family protein |
| Translation | RNA transport | *Glyma.14G023300* | 2.43 | 0.00 | Translation initiation factor SUI1 family protein |
| Translation | mRNA surveillance pathway | *Glyma.02G006000* | 1.02 | 0.00 | type one serine/threonine protein phosphatase 4 |
| Translation | mRNA surveillance pathway | *Glyma.04G086800* | -1.26 | 0.04 | nuclear poly(a) polymerase |
| Translation | mRNA surveillance pathway | *Glyma.06G088500* | -1.24 | 0.04 | nuclear poly(a) polymerase |
| Translation | mRNA surveillance pathway | *Glyma.08G172000* | -1.28 | 0.04 | arginine/serine-rich 45 |
| Translation | mRNA surveillance pathway | *Glyma.09G035800* | -1.53 | 0.04 | homolog of yeast FIP1 |
| Translation | mRNA surveillance pathway | *Glyma.09G153100* | -1.34 | 0.02 | cleavage and polyadenylation specificity factor 160 |
| Translation | mRNA surveillance pathway | *Glyma.15G140800* | -1.90 | 0.00 | homolog of yeast FIP1 |
| Translation | mRNA surveillance pathway | *Glyma.16G203900* | -1.20 | 0.04 | cleavage and polyadenylation specificity factor 160 |
| Translation | mRNA surveillance pathway | *Glyma.17G222400* | -2.05 | 0.02 | Transducin/WD40 repeat-like superfamily protein |
| Translation | mRNA surveillance pathway | *Glyma.18G040400* | -1.21 | 0.03 | ALWAYS EARLY 4 |
| Translation | mRNA surveillance pathway | *Glyma.18G196200* | -1.45 | 0.00 | mRNA capping enzyme family protein |
| Translation | mRNA surveillance pathway | *TCONS_00018646* | -1.37 | 0.01 | PREDICTED: nuclear poly(A) polymerase 4-like isoform X2 |
| Translation | mRNA surveillance pathway | *TCONS_00018648* | -1.28 | 0.02 | PREDICTED: nuclear poly(A) polymerase 4-like isoform X2 |
| Translation | mRNA surveillance pathway | *TCONS_00076188* | -1.78 | 0.04 | Pre-mRNA 3'-end-processing factor FIP1 |
| Translation | mRNA surveillance pathway | *TCONS_00102979* | -1.67 | 0.00 | PREDICTED: polyadenylation and cleavage factor homolog 4-like |
| Translation | mRNA surveillance pathway | *Glyma.09G066700* | 1.20 | 0.02 | homolog of CFIM-25 |
| Translation | mRNA surveillance pathway | *Glyma.10G170200* | 2.61 | 0.00 | Eukaryotic release factor 1 (eRF1) family protein |
| Translation | mRNA surveillance pathway | *Glyma.15G172700* | 2.58 | 0.05 | homolog of CFIM-25 |
| Translation | mRNA surveillance pathway | *Glyma.19G188500* | 1.97 | 0.00 | Eukaryotic release factor 1 (eRF1) family protein |
| Transcription | RNA polymerase | *Glyma.09G066000* | -1.42 | 0.01 | DNA-directed RNA polymerase family protein |
| Transcription | RNA polymerase | *Glyma.10G095200* | -1.72 | 0.00 | nuclear RNA polymerase A1 |
| Transcription | RNA polymerase | *Glyma.11G009900* | -1.21 | 0.04 | SIN-like family protein |
| Transcription | RNA polymerase | *Glyma.13G192600* | -1.75 | 0.00 | nuclear RNA polymerase A2 |
| Transcription | RNA polymerase | *Glyma.15G121800* | -1.35 | 0.04 | RNA polymerase II, Rpb4, core protein |
| Transcription | Basal transcription factors | *Glyma.04G044700* | -2.49 | 0.00 | TBP-associated factor 4 |
| Transcription | Basal transcription factors | *Glyma.09G215000* | -1.27 | 0.02 | HAC13 protein (HAC13) |
| Transcription | Basal transcription factors | *TCONS_00007740* | -1.60 | 0.03 | TATA-binding protein2 isoform 1 |
| Transcription | Basal transcription factors | *TCONS_00086421* | -1.23 | 0.04 | PREDICTED: transcription initiation factor TFIID subunit 4b-like isoform X1 |
| Transcription | Spliceosome | *Glyma.02G205600* | -4.79 | 0.00 | heat shock protein 70B |
| Transcription | Spliceosome | *Glyma.08G173500* | -1.31 | 0.05 | U5 small nuclear ribonucleoprotein helicase, putative |
| Transcription | Spliceosome | *Glyma.09G104200* | -1.89 | 0.01 | SC35-like splicing factor 30A |
| Transcription | Spliceosome | *Glyma.09G114600* | -1.23 | 0.04 |  |
| Transcription | Spliceosome | *Glyma.11G099400* | -8.02 | 0.03 | mRNA splicing factor, thioredoxin-like U5 snRNP |
| Transcription | Spliceosome | *Glyma.12G181300* | -1.53 | 0.01 | Zinc finger C-x8-C-x5-C-x3-H type family protein |
| Transcription | Spliceosome | *Glyma.14G078500* | -1.38 | 0.02 |  |
| Transcription | Spliceosome | *Glyma.16G095600* | -1.20 | 0.05 | RNA recognition motif (RRM)-containing protein |
| Transcription | Spliceosome | *Glyma.17G072400* | -3.23 | 0.01 | heat shock protein 70B |
| Transcription | Spliceosome | *Glyma.17G078400* | -1.42 | 0.02 | glycine-rich RNA-binding protein 3 |
| Transcription | Spliceosome | *Glyma.17G233000* | -1.18 | 0.05 | pre-mRNA-processing protein 40A |
| Transcription | Spliceosome | *Glyma.18G040400* | -1.21 | 0.03 | ALWAYS EARLY 4 |
| Transcription | Spliceosome | *Glyma.18G289100* | -2.34 | 0.03 | heat shock cognate protein 70-1 |
| Transcription | Spliceosome | *Glyma.U029000* | -1.72 | 0.02 | pre-mRNA-processing protein 40A |
| Transcription | Spliceosome | *TCONS_00011666* | -1.40 | 0.04 | PREDICTED: glycine-rich RNA-binding protein 3, mitochondrial |
| Transcription | Spliceosome | *TCONS_00011667* | -1.58 | 0.02 | Glycine-rich RNA-binding protein 2, mitochondrial |
| Transcription | Spliceosome | *TCONS_00062542* | -1.17 | 0.05 | PREDICTED: UBP1-associated protein 2C |
| Transcription | Spliceosome | *TCONS_00087452* | -1.34 | 0.03 | PREDICTED: glycine-rich RNA-binding protein blt801 |
| Transcription | Spliceosome | *Glyma.18G004000* | 1.67 | 0.00 | ATP-dependent RNA helicase, putative |
| Transcription | Spliceosome | *TCONS_00037021* | 1.10 | 0.05 | Serine/arginine-rich splicing factor 6 |

**Tab.** **S4** The DEGs associated with lipid metabolism. (The up regulated genes are colored with red, and the down regulated genes are colored with green.)

| **KEGG pathway name** | **Gene name** | **log_2_FC** | **FDR** | **Description** |
| --- | --- | --- | --- | --- |
| Fatty acid elongation | *Glyma.06G214800* | -1.41 | 0.03 | 3-ketoacyl-CoA synthase 11 |
| Fatty acid elongation | *TCONS_00010591* | 1.88 | 0.00 | Acyl-coenzyme A thioesterase 8, partial |
| Fatty acid elongation | *Glyma.02G001500* | 1.51 | 0.00 | 3-ketoacyl-CoA synthase 11 |
| Fatty acid elongation | *Glyma.02G273300* | 2.96 | 0.00 | 3-oxo-5-alpha-steroid 4-dehydrogenase family protein |
| Fatty acid elongation | *Glyma.03G009800* | 2.37 | 0.00 | Acyl-CoA thioesterase family protein |
| Fatty acid elongation | *Glyma.08G279700* | 3.35 | 0.00 | Protein-tyrosine phosphatase-like, PTPLA |
| Fatty acid elongation | *Glyma.10G001800* | 3.89 | 0.00 | 3-ketoacyl-CoA synthase 11 |
| Fatty acid elongation | *Glyma.10G179400* | 2.05 | 0.00 | 3-ketoacyl-CoA synthase 11 |
| Fatty acid elongation | *Glyma.10G274400* | 3.28 | 0.00 | 3-ketoacyl-CoA synthase 6 |
| Fatty acid elongation | *Glyma.11G245600* | 1.38 | 0.00 | beta-ketoacyl reductase 1 |
| Fatty acid elongation | *Glyma.13G331600* | 1.31 | 0.00 | 3-ketoacyl-CoA synthase 12 |
| Fatty acid elongation | *Glyma.14G043500* | 1.50 | 0.00 | 3-oxo-5-alpha-steroid 4-dehydrogenase family protein |
| Fatty acid elongation | *Glyma.15G042500* | 2.77 | 0.00 | 3-ketoacyl-CoA synthase 12 |
| Fatty acid elongation | *Glyma.18G011600* | 1.89 | 0.00 | beta-ketoacyl reductase 1 |
| Fatty acid elongation | *Glyma.18G146900* | 2.50 | 0.00 | Protein-tyrosine phosphatase-like, PTPLA |
| Fatty acid elongation | *Glyma.20G115500* | 2.66 | 0.00 | 3-ketoacyl-CoA synthase 6 |
| Fatty acid elongation | *Glyma.20G210900* | 1.06 | 0.00 | 3-ketoacyl-CoA synthase 11 |
| Synthesis and degradation of ketone bodies | *TCONS_00004235* | -1.64 | 0.00 | PREDICTED: hydroxymethylglutaryl-CoA synthase |
| Synthesis and degradation of ketone bodies | *TCONS_00054476* | -1.77 | 0.00 | PREDICTED: hydroxymethylglutaryl-CoA synthase |
| Synthesis and degradation of ketone bodies | *TCONS_00054477* | -1.79 | 0.00 | PREDICTED: hydroxymethylglutaryl-CoA synthase |
| Synthesis and degradation of ketone bodies | *Glyma.01G215500* | -1.57 | 0.01 | hydroxymethylglutaryl-CoA synthase / HMG-CoA synthase / 3-hydroxy-3-methylglutaryl coenzyme A synthase |
| Synthesis and degradation of ketone bodies | *Glyma.11G027000* | -1.75 | 0.00 | hydroxymethylglutaryl-CoA synthase / HMG-CoA synthase / 3-hydroxy-3-methylglutaryl coenzyme A synthase |
| Synthesis and degradation of ketone bodies | *Glyma.02G277900* | 1.41 | 0.00 | Aldolase superfamily protein |
| Synthesis and degradation of ketone bodies | *Glyma.16G200200* | 1.28 | 0.00 | hydroxymethylglutaryl-CoA synthase / HMG-CoA synthase / 3-hydroxy-3-methylglutaryl coenzyme A synthase |
| Cutin, suberine and wax biosynthesis | *TCONS_00011356* | -2.31 | 0.00 | Protein WAX2 |
| Cutin, suberine and wax biosynthesis | *Glyma.03G101200* | -2.23 | 0.00 | Fatty acid hydroxylase superfamily |
| Cutin, suberine and wax biosynthesis | *Glyma.03G101300* | -1.73 | 0.03 | Fatty acid hydroxylase superfamily |
| Cutin, suberine and wax biosynthesis | *Glyma.08G193100* | -1.10 | 0.10 | Glucose-methanol-choline (GMC) oxidoreductase family protein |
| Cutin, suberine and wax biosynthesis | *Glyma.11G185100* | -1.61 | 0.01 | Jojoba acyl CoA reductase-related male sterility protein |
| Cutin, suberine and wax biosynthesis | *Glyma.13G350300* | -1.40 | 0.02 | Glucose-methanol-choline (GMC) oxidoreductase family protein |
| Cutin, suberine and wax biosynthesis | *Glyma.15G024200* | -1.51 | 0.01 | Glucose-methanol-choline (GMC) oxidoreductase family protein |
| Cutin, suberine and wax biosynthesis | *Glyma.16G053300* | -1.84 | 0.23 | HXXXD-type acyl-transferase family protein |
| Cutin, suberine and wax biosynthesis | *Glyma.20G002700* | -6.22 | 0.00 | cytochrome P450, family 86, subfamily B, polypeptide 1 |
| Cutin, suberine and wax biosynthesis | *TCONS_00046966* | 1.56 | 0.00 | PREDICTED: probable peroxygenase 5 isoform X1 |
| Cutin, suberine and wax biosynthesis | *Glyma.03G101800* | 8.78 | 0.00 | Fatty acid hydroxylase superfamily |
| Cutin, suberine and wax biosynthesis | *Glyma.07G114200* | 2.37 | 0.00 | Fatty acid hydroxylase superfamily |
| Cutin, suberine and wax biosynthesis | *Glyma.08G010500* | 3.35 | 0.00 | HXXXD-type acyl-transferase family protein |
| Cutin, suberine and wax biosynthesis | *Glyma.09G123800* | 0.80 | 0.02 | Caleosin-related family protein |
| Cutin, suberine and wax biosynthesis | *Glyma.11G185300* | 3.53 | 0.00 | Jojoba acyl CoA reductase-related male sterility protein |
| Cutin, suberine and wax biosynthesis | *Glyma.13G120500* | 1.14 | 0.00 | cytochrome P450, family 94, subfamily B, polypeptide 1 |
| Cutin, suberine and wax biosynthesis | *Glyma.17G217200* | 2.10 | 0.02 | HXXXD-type acyl-transferase family protein |
| Biosynthesis of unsaturated fatty acids | *TCONS_00051112* | -2.48 | 0.00 | omega-6 fatty acid desaturase, endoplasmic reticulum isozyme 1 |
| Biosynthesis of unsaturated fatty acids | *Glyma.07G180100* | -1.48 | 0.01 | peroxisomal 3-ketoacyl-CoA thiolase 3 |
| Biosynthesis of unsaturated fatty acids | *Glyma.10G278000* | -2.30 | 0.00 | fatty acid desaturase 2 |
| Biosynthesis of unsaturated fatty acids | *Glyma.14G121400* | -7.53 | 0.00 | Plant stearoyl-acyl-carrier-protein desaturase family protein |
| Biosynthesis of unsaturated fatty acids | *TCONS_00010591* | 1.88 | 0.00 | Acyl-coenzyme A thioesterase 8, partial |
| Biosynthesis of unsaturated fatty acids | *TCONS_00021958* | 1.36 | 0.01 | Acyl-coenzyme A oxidase 2, peroxisomal |
| Biosynthesis of unsaturated fatty acids | *Glyma.02G273300* | 2.96 | 0.00 | 3-oxo-5-alpha-steroid 4-dehydrogenase family protein |
| Biosynthesis of unsaturated fatty acids | *Glyma.03G009800* | 2.37 | 0.00 | Acyl-CoA thioesterase family protein |
| Biosynthesis of unsaturated fatty acids | *Glyma.05G180100* | 1.32 | 0.01 | acyl-CoA oxidase 2 |
| Biosynthesis of unsaturated fatty acids | *Glyma.07G030000* | 0.85 | 0.06 | fatty acid desaturase 5 |
| Biosynthesis of unsaturated fatty acids | *Glyma.08G279700* | 3.35 | 0.00 | Protein-tyrosine phosphatase-like, PTPLA |
| Biosynthesis of unsaturated fatty acids | *Glyma.11G245600* | 1.38 | 0.00 | beta-ketoacyl reductase 1 |
| Biosynthesis of unsaturated fatty acids | *Glyma.14G043500* | 1.50 | 0.00 | 3-oxo-5-alpha-steroid 4-dehydrogenase family protein |
| Biosynthesis of unsaturated fatty acids | *Glyma.18G011600* | 1.89 | 0.00 | beta-ketoacyl reductase 1 |
| Biosynthesis of unsaturated fatty acids | *Glyma.18G146900* | 2.50 | 0.00 | Protein-tyrosine phosphatase-like, PTPLA |
| Fatty acid degradation | *TCONS_00059051* | -3.46 | 0.00 | PREDICTED: alcohol dehydrogenase-like 2 |
| Fatty acid degradation | *Glyma.02G010300* | -1.25 | 0.02 | AMP-dependent synthetase and ligase family protein |
| Fatty acid degradation | *Glyma.04G240800* | -4.41 | 0.00 | alcohol dehydrogenase 1 |
| Fatty acid degradation | *Glyma.07G180100* | -1.48 | 0.01 | peroxisomal 3-ketoacyl-CoA thiolase 3 |
| Fatty acid degradation | *Glyma.12G015100* | -3.68 | 0.00 | Zinc-binding alcohol dehydrogenase family protein |
| Fatty acid degradation | *Glyma.12G015400* | -3.19 | 0.00 | Zinc-binding alcohol dehydrogenase family protein |
| Fatty acid degradation | *Glyma.13G170600* | -3.00 | 0.03 | aldehyde dehydrogenase 2B4 |
| Fatty acid degradation | *Glyma.14G152100* | -1.27 | 0.04 | aldehyde dehydrogenase 3H1 |
| Fatty acid degradation | *Glyma.17G027600* | -1.42 | 0.04 | multifunctional protein 2 |
| Fatty acid degradation | *Glyma.18G200300* | -1.87 | 0.00 | GroES-like zinc-binding dehydrogenase family protein |
| Fatty acid degradation | *TCONS_00021958* | 1.36 | 0.01 | Acyl-coenzyme A oxidase 2, peroxisomal |
| Fatty acid degradation | *Glyma.02G034000* | 1.09 | 0.04 | aldehyde dehydrogenase 2B7 |
| Fatty acid degradation | *Glyma.03G221400* | 5.97 | 0.00 | AMP-dependent synthetase and ligase family protein |
| Fatty acid degradation | *Glyma.05G180100* | 1.32 | 0.01 | acyl-CoA oxidase 2 |
| Fatty acid degradation | *Glyma.11G133300* | 3.28 | 0.00 | aldehyde dehydrogenase 3F1 |
| Fatty acid degradation | *Glyma.13G340000* | 1.18 | 0.00 | aldehyde dehydrogenase 3F1 |
| Fatty acid degradation | *Glyma.19G218300* | 5.54 | 0.00 | AMP-dependent synthetase and ligase family protein |
| Linoleic acid metabolism | *Glyma.07G007000* | -1.63 | 0.01 | lipoxygenase 1 |
| Linoleic acid metabolism | *Glyma.08G189300* | -3.23 | 0.00 | lipoxygenase 1 |
| Linoleic acid metabolism | *Glyma.08G189600* | -4.15 | 0.00 | lipoxygenase 1 |
| Linoleic acid metabolism | *Glyma.13G347800* | -1.57 | 0.03 | lipoxygenase 1 |
| Linoleic acid metabolism | *TCONS_00013997* | 2.06 | 0.00 | PREDICTED: triacylglycerol lipase SDP1-like |
| Linoleic acid metabolism | *Glyma.03G130900* | 1.65 | 0.00 | Patatin-like phospholipase family protein |
| Linoleic acid metabolism | *Glyma.03G237300* | 1.11 | 0.00 | lipoxygenase 1 |
| Linoleic acid metabolism | *Glyma.07G129900* | 1.70 | 0.00 | Phospholipase A2 family protein |
| Linoleic acid metabolism | *Glyma.13G030300* | 3.97 | 0.01 | lipoxygenase 2 |
| Linoleic acid metabolism | *Glyma.19G132900* | 1.08 | 0.01 | Patatin-like phospholipase family protein |
| Linoleic acid metabolism | *Glyma.20G144600* | 0.64 | 0.55 | PLAT/LH2 domain-containing lipoxygenase family protein |
| alpha-Linolenic acid metabolism | *Glyma.02G053900* | -4.61 | 0.02 | S-adenosyl-L-methionine-dependent methyltransferases superfamily protein |
| alpha-Linolenic acid metabolism | *Glyma.04G240800* | -4.41 | 0.00 | alcohol dehydrogenase 1 |
| alpha-Linolenic acid metabolism | *Glyma.07G162900* | -1.22 | 0.05 | allene oxide synthase |
| alpha-Linolenic acid metabolism | *Glyma.07G180100* | -1.48 | 0.01 | peroxisomal 3-ketoacyl-CoA thiolase 3 |
| alpha-Linolenic acid metabolism | *Glyma.09G254200* | -7.69 | 0.00 | jasmonic acid carboxyl methyltransferase |
| alpha-Linolenic acid metabolism | *Glyma.17G027600* | -1.42 | 0.04 | multifunctional protein 2 |
| alpha-Linolenic acid metabolism | *TCONS_00013997* | 2.06 | 0.00 | PREDICTED: triacylglycerol lipase SDP1-like |
| alpha-Linolenic acid metabolism | *TCONS_00021958* | 1.36 | 0.01 | Acyl-coenzyme A oxidase 2, peroxisomal |
| alpha-Linolenic acid metabolism | *TCONS_00086155* | 3.28 | 0.00 | 12-oxophytodienoate reductase 1 |
| alpha-Linolenic acid metabolism | *Glyma.03G130900* | 1.65 | 0.00 | Patatin-like phospholipase family protein |
| alpha-Linolenic acid metabolism | *Glyma.04G035000* | 3.57 | 0.02 | allene oxide synthase |
| alpha-Linolenic acid metabolism | *Glyma.05G180100* | 1.32 | 0.01 | acyl-CoA oxidase 2 |
| alpha-Linolenic acid metabolism | *Glyma.07G129900* | 1.70 | 0.00 | Phospholipase A2 family protein |
| alpha-Linolenic acid metabolism | *Glyma.08G256600* | 1.22 | 0.00 | allene oxide cyclase 4 |
| alpha-Linolenic acid metabolism | *Glyma.13G030300* | 3.97 | 0.01 | lipoxygenase 2 |
| alpha-Linolenic acid metabolism | *Glyma.14G223600* | 3.60 | 0.00 | 12-oxophytodienoate reductase 1 |
| alpha-Linolenic acid metabolism | *Glyma.15G223900* | 7.16 | 0.03 | 12-oxophytodienoate reductase 2 |
| alpha-Linolenic acid metabolism | *Glyma.17G209900* | 3.49 | 0.00 | 12-oxophytodienoate reductase 1 |
| alpha-Linolenic acid metabolism | *Glyma.19G057500* | 1.14 | 0.00 | 12-oxophytodienoate reductase 2 |
| alpha-Linolenic acid metabolism | *Glyma.19G132900* | 1.08 | 0.01 | Patatin-like phospholipase family protein |
| Fatty acid biosynthesis | *TCONS_00058131* | -1.69 | 0.03 | PREDICTED: somatic embryogenesis receptor kinase 1-like isoform X3 |
| Fatty acid biosynthesis | *Glyma.02G010300* | -1.25 | 0.02 | AMP-dependent synthetase and ligase family protein |
| Fatty acid biosynthesis | *Glyma.05G218600* | -1.35 | 0.04 | 3-ketoacyl-acyl carrier protein synthase I |
| Fatty acid biosynthesis | *Glyma.10G268200* | -4.64 | 0.00 | fatty acyl-ACP thioesterases B |
| Fatty acid biosynthesis | *Glyma.14G121400* | -7.53 | 0.00 | Plant stearoyl-acyl-carrier-protein desaturase family protein |
| Fatty acid biosynthesis | *TCONS_00019207* | 2.11 | 0.00 | PREDICTED: palmitoyl-acyl carrier protein thioesterase, chloroplastic-like |
| Fatty acid biosynthesis | *TCONS_00019208* | 2.10 | 0.00 | PREDICTED: palmitoyl-acyl carrier protein thioesterase, chloroplastic-like |
| Fatty acid biosynthesis | *Glyma.03G221400* | 5.97 | 0.00 | AMP-dependent synthetase and ligase family protein |
| Fatty acid biosynthesis | *Glyma.04G151600* | 1.97 | 0.00 | fatty acyl-ACP thioesterases B |
| Fatty acid biosynthesis | *Glyma.19G218300* | 5.54 | 0.00 | AMP-dependent synthetase and ligase family protein |
| Steroid biosynthesis | *Glyma.08G167700* | -2.62 | 0.00 | Myzus persicae-induced lipase 1 |
| Steroid biosynthesis | *Glyma.09G186900* | -2.03 | 0.00 | C-8,7 sterol isomerase |
| Steroid biosynthesis | *Glyma.18G118100* | -1.26 | 0.04 | FAD/NAD(P)-binding oxidoreductase family protein |
| Steroid biosynthesis | *TCONS_00013997* | 2.06 | 0.00 | PREDICTED: triacylglycerol lipase SDP1-like |
| Steroid biosynthesis | *Glyma.03G130900* | 1.65 | 0.00 | Patatin-like phospholipase family protein |
| Steroid biosynthesis | *Glyma.13G003000* | 5.43 | 0.00 | Myzus persicae-induced lipase 1 |
| Steroid biosynthesis | *Glyma.13G253100* | 1.33 | 0.01 | FAD/NAD(P)-binding oxidoreductase family protein |
| Steroid biosynthesis | *Glyma.15G061800* | 1.98 | 0.00 | FAD/NAD(P)-binding oxidoreductase family protein |
| Steroid biosynthesis | *Glyma.15G276300* | 1.27 | 0.00 | sterol methyltransferase 1 |
| Steroid biosynthesis | *Glyma.19G132900* | 1.08 | 0.01 | Patatin-like phospholipase family protein |
| Steroid biosynthesis | *Glyma.20G192700* | 4.91 | 0.11 | cycloartenol synthase 1 |
| Arachidonic acid metabolism | *Glyma.07G126200* | -1.65 | 0.03 | gamma-glutamyl transpeptidase 4 |
| Arachidonic acid metabolism | *TCONS_00001875* | 1.04 | 0.00 | PREDICTED: probable glutathione peroxidase 8 |
| Arachidonic acid metabolism | *TCONS_00013997* | 2.06 | 0.00 | PREDICTED: triacylglycerol lipase SDP1-like |
| Arachidonic acid metabolism | *Glyma.03G130900* | 1.65 | 0.00 | Patatin-like phospholipase family protein |
| Arachidonic acid metabolism | *Glyma.07G129900* | 1.70 | 0.00 | Phospholipase A2 family protein |
| Arachidonic acid metabolism | *Glyma.19G132900* | 1.08 | 0.01 | Patatin-like phospholipase family protein |
| Ether lipid metabolism | *Glyma.04G020400* | -2.12 | 0.00 | phospholipase D delta |
| Ether lipid metabolism | *Glyma.07G031100* | -1.28 | 0.04 | phospholipase D alpha 1 |
| Ether lipid metabolism | *TCONS_00013997* | 2.06 | 0.00 | PREDICTED: triacylglycerol lipase SDP1-like |
| Ether lipid metabolism | *TCONS_00024232* | 1.04 | 0.03 | PREDICTED: phospholipase D delta-like |
| Ether lipid metabolism | *TCONS_00028687* | 1.86 | 0.00 | PREDICTED: phospholipase D alpha 1-like |
| Ether lipid metabolism | *Glyma.03G130900* | 1.65 | 0.00 | Patatin-like phospholipase family protein |
| Ether lipid metabolism | *Glyma.06G068600* | 2.26 | 0.00 | phospholipase D alpha 1 |
| Ether lipid metabolism | *Glyma.07G129900* | 1.70 | 0.00 | Phospholipase A2 family protein |
| Ether lipid metabolism | *Glyma.15G023500* | 1.28 | 0.00 | phospholipase D alpha 4 |
| Ether lipid metabolism | *Glyma.19G132900* | 1.08 | 0.01 | Patatin-like phospholipase family protein |
| Glycerolipid metabolism | *Glyma.11G190400* | -2.53 | 0.03 | Lecithin: cholesterol acyltransferase family protein |
| Glycerolipid metabolism | *Glyma.13G170600* | -3.00 | 0.03 | aldehyde dehydrogenase 2B4 |
| Glycerolipid metabolism | *Glyma.14G028300* | -1.93 | 0.00 | glycerol-3-phosphate acyltransferase 1 |
| Glycerolipid metabolism | *Glyma.14G152100* | -1.27 | 0.04 | aldehyde dehydrogenase 3H1 |
| Glycerolipid metabolism | *Glyma.15G034100* | -2.15 | 0.06 | lysophosphatidyl acyltransferase 2 |
| Glycerolipid metabolism | *Glyma.17G242900* | -0.68 | 1.00 | phosphatidic acid phosphohydrolase 2 |
| Glycerolipid metabolism | *Glyma.18G107100* | -4.78 | 0.00 | glycerol-3-phosphate acyltransferase 1 |
| Glycerolipid metabolism | *TCONS_00013997* | 2.06 | 0.00 | PREDICTED: triacylglycerol lipase SDP1-like |
| Glycerolipid metabolism | *Glyma.02G010600* | 2.71 | 0.00 | glycerol-3-phosphate acyltransferase 3 |
| Glycerolipid metabolism | *Glyma.02G034000* | 1.09 | 0.04 | aldehyde dehydrogenase 2B7 |
| Glycerolipid metabolism | *Glyma.03G130900* | 1.65 | 0.00 | Patatin-like phospholipase family protein |
| Glycerolipid metabolism | *Glyma.03G221300* | 3.18 | 0.00 | glycerol-3-phosphate acyltransferase 2 |
| Glycerolipid metabolism | *Glyma.05G196100* | 1.34 | 0.00 | diacylglycerol kinase 2 |
| Glycerolipid metabolism | *Glyma.09G160400* | 2.69 | 0.00 | alpha-galactosidase 2 |
| Glycerolipid metabolism | *Glyma.10G011000* | 3.26 | 0.00 | glycerol-3-phosphate acyltransferase 3 |
| Glycerolipid metabolism | *Glyma.11G133300* | 3.28 | 0.00 | aldehyde dehydrogenase 3F1 |
| Glycerolipid metabolism | *Glyma.13G085700* | 3.25 | 0.00 | glycerol-3-phosphate acyltransferase 3 |
| Glycerolipid metabolism | *Glyma.13G340000* | 1.18 | 0.00 | aldehyde dehydrogenase 3F1 |
| Glycerolipid metabolism | *Glyma.16G209800* | 1.58 | 0.00 | alpha-galactosidase 2 |
| Glycerolipid metabolism | *Glyma.19G132900* | 1.08 | 0.01 | Patatin-like phospholipase family protein |
| Sphingolipid metabolism | *Glyma.15G072800* | -1.36 | 0.02 | fatty acid desaturase family protein |
| Sphingolipid metabolism | *Glyma.08G058400* | 1.55 | 0.03 | sphingosine kinase 1 |
| Sphingolipid metabolism | *Glyma.09G160400* | 2.69 | 0.00 | alpha-galactosidase 2 |
| Sphingolipid metabolism | *Glyma.16G209800* | 1.58 | 0.00 | alpha-galactosidase 2 |
| Glycerophospholipid metabolism | *TCONS_00021329* | -1.33 | 0.04 | Glycerol-3-phosphate dehydrogenase |
| Glycerophospholipid metabolism | *Glyma.04G020400* | -2.12 | 0.00 | phospholipase D delta |
| Glycerophospholipid metabolism | *Glyma.07G031100* | -1.28 | 0.04 | phospholipase D alpha 1 |
| Glycerophospholipid metabolism | *Glyma.11G148900* | -1.23 | 0.04 | 6-phosphogluconate dehydrogenase family protein |
| Glycerophospholipid metabolism | *Glyma.14G028300* | -1.93 | 0.00 | glycerol-3-phosphate acyltransferase 1 |
| Glycerophospholipid metabolism | *Glyma.15G034100* | -2.15 | 0.06 | lysophosphatidyl acyltransferase 2 |
| Glycerophospholipid metabolism | *Glyma.17G242900* | -0.68 | 1.00 | phosphatidic acid phosphohydrolase 2 |
| Glycerophospholipid metabolism | *Glyma.18G107100* | -4.78 | 0.00 | glycerol-3-phosphate acyltransferase 1 |
| Glycerophospholipid metabolism | *Glyma.19G098500* | -2.37 | 0.00 | senescence-related gene 3 |
| Glycerophospholipid metabolism | *TCONS_00013997* | 2.06 | 0.00 | PREDICTED: triacylglycerol lipase SDP1-like |
| Glycerophospholipid metabolism | *TCONS_00024232* | 1.04 | 0.03 | PREDICTED: phospholipase D delta-like |
| Glycerophospholipid metabolism | *TCONS_00028687* | 1.86 | 0.00 | PREDICTED: phospholipase D alpha 1-like |
| Glycerophospholipid metabolism | *Glyma.02G010600* | 2.71 | 0.00 | glycerol-3-phosphate acyltransferase 3 |
| Glycerophospholipid metabolism | *Glyma.03G130900* | 1.65 | 0.00 | Patatin-like phospholipase family protein |
| Glycerophospholipid metabolism | *Glyma.03G221300* | 3.18 | 0.00 | glycerol-3-phosphate acyltransferase 2 |
| Glycerophospholipid metabolism | *Glyma.05G196100* | 1.34 | 0.00 | diacylglycerol kinase 2 |
| Glycerophospholipid metabolism | *Glyma.06G068600* | 2.26 | 0.00 | phospholipase D alpha 1 |
| Glycerophospholipid metabolism | *Glyma.07G129900* | 1.70 | 0.00 | Phospholipase A2 family protein |
| Glycerophospholipid metabolism | *Glyma.10G011000* | 3.26 | 0.00 | glycerol-3-phosphate acyltransferase 3 |
| Glycerophospholipid metabolism | *Glyma.13G085700* | 3.25 | 0.00 | glycerol-3-phosphate acyltransferase 3 |
| Glycerophospholipid metabolism | *Glyma.15G023500* | 1.28 | 0.00 | phospholipase D alpha 4 |
| Glycerophospholipid metabolism | *Glyma.16G052000* | 1.16 | 0.02 | PLC-like phosphodiesterases superfamily protein |
| Glycerophospholipid metabolism | *Glyma.19G132900* | 1.08 | 0.01 | Patatin-like phospholipase family protein |

**Tab.** **S5** Expression level of genes associated with the actin filament assembly. (The differential expressed genes are highlighted with yellow color)

| **Protein name** | **Gene name** | ***Gmdtm1-1* (FPKM-value)** | | | **Williams 82 (FPKM-value)** | | | **homologous gene in Arabidopsis** | **Description** |
| --- | --- | --- | --- | --- | --- | --- | --- | --- | --- |
|  |  | **Repeat1** | **Repeat2** | **Repeat3** | **Repeat1** | **Repeat2** | **Repeat3** |  |  |
| RAC protein | *Glyma.01G164200* | 5.15 | 3.76 | 3.97 | 4.55 | 6.57 | 4.80 | *AT3G51300.1* | RHO-related protein from plants 1 |
|  | *Glyma.02G046100* | 0.00 | 0.00 | 0.00 | 0.00 | 0.00 | 0.00 | *AT4G35950.1* | RAC-like 6 |
|  | *Glyma.04G023300* | 5.49 | 2.62 | 3.27 | 5.72 | 6.99 | 6.33 | *AT4G35020.1* | RAC-like 3 |
|  | *Glyma.04G023400* | 7.25 | 6.62 | 6.18 | 8.76 | 9.21 | 11.96 | *AT4G35020.1* | RAC-like 3 |
|  | *Glyma.04G180200* | 2.11 | 1.48 | 2.00 | 1.81 | 1.44 | 1.93 | *AT3G48040.1* | RHO-related protein from plants 10 |
|  | *Glyma.05G035200* | 2.68 | 2.26 | 3.13 | 1.56 | 1.16 | 1.30 | *AT3G48040.1* | RHO-related protein from plants 10 |
|  | *Glyma.06G023300* | 9.25 | 6.36 | 5.52 | 9.49 | 13.14 | 15.06 | *AT4G35020.1* | RAC-like 3 |
|  | *Glyma.06G184500* | 3.61 | 2.29 | 3.25 | 3.17 | 3.06 | 3.52 | *AT3G48040.1* | RHO-related protein from plants 10 |
|  | *Glyma.07G084200* | 7.93 | 2.54 | 3.24 | 1.91 | 6.93 | 2.69 | *AT4G28950.1* | RHO-related protein from plants 9 |
|  | *Glyma.07G203100* | 3.25 | 1.48 | 2.94 | 0.89 | 0.35 | 0.38 | *AT5G45970.1* | RAC-like 2 |
|  | *Glyma.09G192700* | 1.82 | 0.50 | 0.79 | 1.03 | 2.28 | 1.18 | *AT4G28950.1* | RHO-related protein from plants 9 |
|  | *Glyma.11G079100* | 7.89 | 5.36 | 5.72 | 7.63 | 10.12 | 7.55 | *AT3G51300.1* | RHO-related protein from plants 1 |
|  | *Glyma.11G107700* | 21.45 | 17.36 | 19.27 | 28.57 | 29.47 | 36.44 | *AT4G35020.1* | RAC-like 3 |
|  | *Glyma.12G032700* | 20.06 | 19.48 | 19.43 | 22.76 | 27.98 | 24.57 | *AT4G35020.1* | RAC-like 3 |
|  | *Glyma.12G120600* | 3.46 | 0.76 | 1.31 | 2.59 | 5.97 | 3.72 | *AT4G35020.1* | RAC-like 3 |
|  | *Glyma.12G208000* | 0.13 | 0.11 | 0.11 | 0.04 | 0.02 | 0.02 | *AT1G75840.1* | RAC-like GTP binding protein 5 |
|  | *Glyma.13G172600* | 0.06 | 0.00 | 0.17 | 0.03 | 0.03 | 0.06 | *AT5G45970.1* | RAC-like 2 |
|  | *Glyma.13G293100* | 0.42 | 0.18 | 0.38 | 0.34 | 0.34 | 0.36 | *AT1G75840.1* | RAC-like GTP binding protein 5 |
|  | *Glyma.16G124400* | 0.00 | 0.00 | 0.02 | 0.00 | 0.02 | 0.02 | *AT4G35950.1* | RAC-like 6 |
|  | *Glyma.17G092100* | 0.24 | 0.22 | 0.53 | 0.20 | 0.10 | 0.21 | *AT3G48040.1* | RHO-related protein from plants 10 |
| WAVE/SCAR | *Glyma.07G221000* | 0.00 | 0.00 | 0.00 | 0.00 | 0.00 | 0.00 | *AT2G35110.2* | NCK-associated protein 1 |
|  | *Glyma.20G019300* | 1.01 | 0.60 | 0.53 | 4.81 | 7.43 | 6.26 | *AT2G35110.2* | NCK-associated protein 1 |
|  | *Glyma.01G050600* | 0.00 | 0.00 | 0.00 | 0.00 | 0.00 | 0.00 | *AT5G18410.1* | SRA1, PIR121 |
|  | *Glyma.03G253000* | 1.21 | 0.61 | 0.82 | 1.10 | 1.95 | 1.43 | *AT5G18410.2* | SRA1, PIR121 |
|  | *Glyma.10G134000* | 0.00 | 0.00 | 0.00 | 0.00 | 0.00 | 0.00 | *AT5G18410.1* | SRA1, PIR121 |
|  | *Glyma.19G250600* | 9.95 | 5.63 | 6.39 | 9.35 | 15.42 | 12.62 | *AT5G18410.2* | SRA1, PIR121 |
|  | *Glyma.01G020200* | 6.07 | 6.39 | 6.41 | 5.82 | 7.21 | 10.18 | *AT2G22640.1* | BRICK1, putative |
|  | *Glyma.09G202200* | 4.19 | 2.50 | 2.99 | 4.51 | 8.18 | 9.38 | *AT2G22640.1* | BRICK1, putative |
|  | *Glyma.01G128700* | 2.52 | 1.33 | 1.42 | 2.37 | 3.49 | 3.03 | *AT2G38440.1* | SCAR homolog 2 |
|  | *Glyma.03G041600* | 1.87 | 0.95 | 1.14 | 1.75 | 2.93 | 2.67 | *AT2G38440.1* | SCAR homolog 2 |
|  | *Glyma.09G279900* | 0.51 | 0.21 | 0.21 | 0.44 | 0.92 | 0.62 | *AT2G38440.1* | SCAR homolog 2 |
|  | *Glyma.18G209100* | 0.73 | 0.30 | 0.32 | 0.56 | 1.04 | 0.71 | *AT2G38440.1* | SCAR homolog 2 |
|  | *Glyma.05G158300* | 1.59 | 0.84 | 0.67 | 1.33 | 1.53 | 1.54 | *AT1G29170.1* | SCAR family protein |
|  | *Glyma.08G116000* | 0.69 | 0.28 | 0.26 | 0.71 | 1.24 | 1.39 | *AT1G29170.1* | SCAR family protein |
|  | *Glyma.03G254100* | 0.02 | 0.04 | 0.11 | 0.05 | 0.08 | 0.04 | *AT2G46225.1* | ABI-1-like 1 |
|  | *Glyma.05G230200* | 0.03 | 0.03 | 0.00 | 0.00 | 0.00 | 0.00 | *AT2G46225.1* | ABI-1-like 1 |
|  | *Glyma.07G062200* | 3.98 | 2.07 | 2.95 | 2.66 | 4.45 | 3.07 | *AT2G46225.1* | ABI-1-like 1 |
|  | *Glyma.08G037700* | 0.00 | 0.00 | 0.07 | 0.00 | 0.04 | 0.00 | *AT2G46225.1* | ABI-1-like 1 |
|  | *Glyma.09G191800* | 8.73 | 3.73 | 4.02 | 4.83 | 2.71 | 3.08 | *AT2G46225.2* | ABI-1-like 1 |
|  | *Glyma.16G030200* | 6.70 | 4.75 | 4.95 | 7.08 | 7.19 | 6.72 | *AT2G46225.1* | ABI-1-like 1 |
|  | *Glyma.19G251600* | 0.12 | 0.07 | 0.17 | 0.02 | 0.08 | 0.02 | *AT2G46225.1* | ABI-1-like 1 |
|  | *Glyma.03G066300* | 0.31 | 0.19 | 0.23 | 0.39 | 0.20 | 0.28 | *AT3G49290.1* | ABL interactor-like protein 2 |
|  | *Glyma.04G234500* | 0.44 | 0.15 | 0.11 | 0.41 | 0.50 | 0.32 | *AT3G49290.1* | ABL interactor-like protein 2 |
|  | *Glyma.05G123100* | 1.15 | 0.61 | 0.35 | 0.78 | 0.49 | 0.59 | *AT3G49290.1* | ABL interactor-like protein 2 |
|  | *Glyma.06G130100* | 0.28 | 0.21 | 0.29 | 0.50 | 0.38 | 0.41 | *AT3G49290.1* | ABL interactor-like protein 2 |
|  | *Glyma.08G037700* | 0.00 | 0.00 | 0.07 | 0.00 | 0.04 | 0.00 | *AT2G46225.1* | ABI-1-like 1 |
|  | *Glyma.03G044700* | 0.00 | 0.00 | 0.00 | 0.00 | 0.00 | 0.00 | *AT5G24310.1* | ABL interactor-like protein 3 |
| ARP2/3 | *Glyma.16G053400* | 3.32 | 2.26 | 1.88 | 2.62 | 3.99 | 2.86 | *AT3G27000.1* | actin related protein 2 |
|  | *Glyma.19G095900* | 2.55 | 2.11 | 2.05 | 2.89 | 4.47 | 3.17 | *AT3G27000.1* | actin related protein 2 |
|  | *Glyma.05G172200* | 2.26 | 1.00 | 1.51 | 2.85 | 3.15 | 2.45 | *AT1G13180.1* | actin related protein 3 |
|  | *Glyma.05G172600* | 8.16 | 4.03 | 3.72 | 8.06 | 9.46 | 6.33 | *AT1G13180.1* | actin related protein 3 |
|  | *Glyma.08G311700* | 0.23 | 0.27 | 0.23 | 0.26 | 0.65 | 0.48 | *AT2G31300.1* | actin-related protein C1B |
|  | *Glyma.10G216300* | 0.61 | 0.28 | 0.22 | 0.36 | 0.73 | 0.40 | *AT2G31300.1* | actin-related protein C1B |
|  | *Glyma.18G103800* | 4.38 | 3.09 | 2.53 | 5.27 | 6.44 | 5.87 | *AT2G31300.1* | actin-related protein C1B |
|  | *Glyma.08G264400* | 1.02 | 0.51 | 0.43 | 1.03 | 2.02 | 1.49 | *AT1G30825.1* | actin-related protein C2A |
|  | *Glyma.16G094500* | 1.36 | 0.37 | 0.73 | 1.15 | 1.74 | 1.21 | *AT1G30825.1* | actin-related protein C2A |
|  | *Glyma.10G136400* | 0.06 | 0.00 | 0.02 | 0.02 | 0.04 | 0.02 | *AT2G33385.2* | actin-related protein C2B |
|  | *Glyma.20G086100* | 0.00 | 0.00 | 0.00 | 0.00 | 0.00 | 0.00 | *AT2G33385.1* | actin-related protein C2B |
|  | *Glyma.13G162500* | 2.48 | 1.99 | 2.20 | 2.18 | 2.63 | 2.22 | *AT1G60430.1* | actin-related protein C3 |
|  | *Glyma.13G162500* | 2.48 | 1.99 | 2.20 | 2.18 | 2.63 | 2.22 | *AT1G60430.1* | actin-related protein C3 |
|  | *Glyma.17G108800* | 1.81 | 1.20 | 1.26 | 1.32 | 1.34 | 1.22 | *AT1G60430.1* | actin-related protein C3 |
|  | *Glyma.01G145800* | 0.00 | 0.00 | 0.00 | 0.00 | 0.00 | 0.00 | *AT4G14147.1* | actin-related protein C4 |
|  | *Glyma.05G153800* | 0.80 | 0.49 | 0.88 | 1.10 | 1.50 | 1.24 | *AT4G14147.2* | actin-related protein C4 |
|  | *Glyma.08G111500* | 2.71 | 0.82 | 1.22 | 1.92 | 3.22 | 2.40 | *AT4G14147.1* | actin-related protein C4 |
|  | *Glyma.03G114200* | 4.18 | 2.75 | 2.36 | 2.78 | 3.22 | 2.30 | *AT4G01710.1* | actin-related protein C5 |
|  | *Glyma.07G112500* | 4.19 | 3.66 | 3.10 | 2.64 | 2.05 | 2.59 | *AT4G01710.1* | actin-related protein C5 |

**Tab. S6** Primers used in this study.

| Primers | Application | Forward sequence 5'-3' | Reverse sequence 5'-3' |
| --- | --- | --- | --- |
| MOL0675 | Marker for mapping | TCCAACAAAAGAATGTGCCCCAAAA | ATTCCGCATGACGCCATCCAATT |
| MOL1169 | Marker for mapping | AAAACGAATCAACGATAGGGTG | GCTATTTAGGCTACCAACCTTTATG |
| MOL2857 | Marker for mapping | TCTTTCAAACTCGGTTCATGTCT | AATGAAGTTGCGCAGATGACA |
| MOL2859 | Marker for mapping | CTTCAGATATAAGAGATTCTGACGATG | CTCAACTCCTAAGGGATGTAGGAT |
| MOL2861 | Marker for mapping | GGTGATACTTAATGCGATTGCTAT | CAAATTAGTCTCTATGTGTTAGGAATGT |
| MOL2867 | Marker for mapping | GCCAAACAAATACGAGACACCT | ATACTTATGGGTGAGGAGATTGAGT |
| OL6754 | Marker for mapping | ATGTCCTCGAGGTTGATGGAG | GTGACAGCCGGAGAAACCTAC |
| OL6756 | Marker for mapping | GAGTTGAATGTGAACCATCTATGC | TATTTCCAAACTGAATGCCAGTG |
| OL6758 | Marker for mapping | AGCATATAAGCATGCGACTTCAG | ACACCCTCGGTGACCAAGTTTAG |
| OL6760 | Marker for mapping | AAGAGTAGGTGGATTGCATGTTG | TCTGGTTGCAATGACTACACTCAC |
| OL6762 | Marker for mapping | ATTGTCTGATTCGGTATCATGTTAC | ATGTCGGCGCTTGGTTAACT |
| OL6786 | Marker for mapping | CCTCCTCGAGTCACTCTGTGGT | CCTTGTCGACAAGCATTATCCA |
| OL6788 | Marker for mapping | CATCCAATCTATTAGCGGGTGT | GGTTAAACAGGGTGTGCCTAAGT |
| OL7131 | To applified *Gmdtm1* promoter | GACTAGTCATACTCTAGTTGGTAAGCAATC | CAGTCTAGGT TCGACTCTTACC |
| OL7133 | To applified *Gmdtm1* CDS | CTGCGACTACGGCCAATTAGTTC | AGGGGGGCCCCCCAGACTTCAAGCTGCGAGATGT |
| OL6830 | To applified *Gmdtm1* CDS | ACACTAACAATAATATCCGCGCTG | CAGCTTCTAACATTTTGATAGAACCA |
| OL6832 | To applified *Gmdtm1* CDS | TGAATCTATCATGGGAGGATTGG | ACAGACTTCAAGCTGCGAGATGT |
| OL6999 | seqence for *Gmdtm1* CDS | GAATCTGCGTGTTGCTCTCTAAG |  |
| OL7000 | seqence for *Gmdtm1* CDS | GTGAGCCCTCGTATTGGAGAG |  |
| OL7001 | seqence for *Gmdtm1* CDS | GCTTTAGCCCAATGTGAAGTGA |  |
| OL7002 | seqence for *Gmdtm1* CDS | AGGATCGGCCACTGAATCTG |  |
| OL7003 | seqence for *Gmdtm1* CDS | TAAGCATCTACCTGATGGAGTACC |  |

**Tab. S7** The primers used for real-time PCR.

| Primers | Gene name | Product length (bp) | PCR efficiency | Forward primer | Reverse primer |
| --- | --- | --- | --- | --- | --- |
| OL8528 | *Glyma.02G001500.1* | 150 | 82.57% | AACCATACATCCCAGATTTCAAG | TGTTACCAAACCTATTTAGTGTCATC |
| OL8530 | *Glyma.02G273300.1* | 213 | 89.54% | ACAACTGAGATCTATCAGTGGCTG | TTGCTTAGGCTTCTTCTACAGGA |
| OL8532 | *Glyma.03G009800.1* | 161 | 94.77% | TGGCATACGCTTCCGATCTA | GGCACTTGGAGTAAAGATCGC |
| OL8534 | *Glyma.08G279700.1* | 200 | 94.51% | TTGGGTCAAACATAGGATATACACA | ATAGCAGATGACTAAGAACCAACAAT |
| OL8536 | *Glyma.10G001800.1* | 113 | 89.67% | TTCAATGAAGGAGCTTGTAAGACTAT | GACGGCTCATGAAGTAGAGTGTG |
| OL8538 | *Glyma.10G179400.1* | 281 | 90.70% | GTTCCTAAGTTAAACAACCTCTTGC | CCCTAAGCTTGATGAGCAACTT |
| OL8540 | *Glyma.10G274400.1* | 190 | 89.10% | AAATAGATGAGTTCTCATAACCCTT | GGCGATCGATTTCTGTGCTAA |
| OL8542 | *Glyma.11G245600.1* | 263 | 100.84% | GGTTCTGCTGTGTAGGCAAATAG | AAGGAAGGAATGACACCAGAGAA |
| OL8544 | *Glyma.13G331600.1* | 212 | 95.03% | TAAGGTCTCCTAATTACAGGCATCA | AAAGTTATTATCACATCCTATTGAAACA |
| OL8546 | *Glyma.15G042500.1* | 167 | 102.26% | TTTTTAGGTCTCCTATAACAGGCATT | AACCACAATTCCCTTTTAACAGAGT |
| OL8548 | *Glyma.18G011600.1* | 153 | 91.22% | TTGGGCTGTGGCATTCTCAT | AGTAGCGTCTATTGGCCTACACAT |
| OL8550 | *Glyma.18G146900.1* | 158 | 90.56% | CAATATGTTAGAGAATTGGTTGGAC | ATGAAATGCGGAGCTCTTTATG |
| OL8556 | *Glyma.20G210900.1* | 184 | 96.37% | TTGGTTCCTAAGTTAAAACCTCTTG | TCGTGTAATAAATGTTCTCATTGGAC |
| OL8558 | *Glyma.03G101200.1* | 144 | 94.69% | CTCTTGCCTACACATTGTGTCAAC | CTACTAACCAAGTCGTCTGTGTGC |
| OL8562 | *Glyma.08G193100.1* | 169 | 90.35% | CGTTGACAGACTTCGTGTGGTAG | CATGATCTTCACACAAGGGATTC |
| OL8564 | *Glyma.11G185100.1* | 144 | 100.25% | AACAAGAATCTCCCCATGTTAGTG | GTGTTTAAGAGTTATGCAATGAGCC |
| OL8566 | *Glyma.13G350300.1* | 164 | 93.54% | ATTCGAAGACAAGAAGGACTGTTAG | TACAGTTTAAACCCAGCTTTGAGGA |
| OL8568 | *Glyma.15G024200.1* | 187 | 105.63% | ATATTAGTTATAGCCCGTGGGACTG | CTAACTGTGGCACGCTAAAACTC |
| OL8574 | *Glyma.20G019300.1* | 171 | 90.18% | CTTCATTTACAGAGCCTGGAACC | CACAAAGGAACAGAATAGATCCAT |
| OL6553 | *Glyma.12G020500.1* | 106 | 96.45% | GATCAGCAATTATGCACAACG | CCGCCACCATTCAGATTATGT |
